# Accelerating Active Learning for Image Classification Through FPGA-Based Implementation

**DOI:** 10.3390/s26123743

**Published:** 2026-06-12

**Authors:** Angelo Barbieri, Christopher A. Flores, Wladimir Valenzuela, Francisco Saavedra

**Affiliations:** 1 Department of Electrical Engineering, Universidad de Concepción, Concepción 4070386, Chilefrsaavedra@udec.cl (F.S.); 2 Department of Electrical and Electronic Engineering, Universidad del Bío-Bío, Concepción 4051381, Chile; 3 Independent Researcher, Concepción 4070386, Chile

**Keywords:** active learning, embedded sensors, FPGA acceleration, image classification, margin sampling, top-*k* selection

## Abstract

Image sensors produce high-dimensional visual data for classification algorithms. Deep Neural Networks (DNNs) achieve high accuracy but require large labeled datasets and computational and energy resources, limiting their use in embedded systems. Active Learning (ALrn) can reduce labeling effort by selecting samples based on informativeness scores, but it remains computationally expensive, especially for high-dimensional images. This work presents a hardware-accelerated approach for the instance selection stage based on a query strategy in uncertainty-based ALrn for image classification using a novel in-line top-k selection algorithm that avoids conventional sorting and reduces memory and computational requirements. The algorithm is implemented on an Xilinx ZYNQ-7000 System on Chip (SoC) using a Field Programmable Gate Array (FPGA)-based accelerator operating at 110 MHz, interfacing with an embedded Advanced RISC Machine (ARM) processor for data acquisition and communication via the Python Productivity for Zynq (PYNQ) framework. Experiments on diverse multiclass datasets demonstrate correctness within an ALrn setting, showing negligible performance deviation in the learning curves compared to software baselines. The accelerator achieves speedup of 231.7× and 22.9× over software baseline and optimized software implementation of the proposed algorithm, respectively, in query-strategy computation while consuming only 0.473 W, substantially lower than conventional Central Processing Unit (CPU)- and Graphics Processing Unit (GPU)-based platforms. These results demonstrate the efficiency and extensibility of the proposed accelerator across alternative ALrn designs and hardware platforms, where the computational cost of instance selection scales with the size of the unlabeled pool.

## 1. Introduction

The proliferation of modern image sensors has transformed them into a significant source of visual data, driving a substantial increase in the volume of information generated over the last decade [1,2]. One example of this is the cameras built into smartphones, whose widespread use has democratized image capture and transmission. Similarly, vision systems in autonomous vehicles use multiple low-cost, high-resolution cameras to perceive the environment and detect objects with great precision [3,4]. In the medical field, imaging devices incorporate specialized sensors that capture visual data for more accurate diagnoses and real-time patient monitoring [5]. In the context of precision agriculture, computer vision systems automatically detect and classify weeds and crops using Deep Learning (DL) models, enabling targeted actions and promoting more efficient and sustainable agricultural practices [6]. Faced with this enormous flow of visual information from different sources, image classification has emerged as a fundamental technique for efficiently organizing, analyzing, and extracting knowledge from the data captured by image sensors [7,8].

Image classification is the task of assigning a label from a predefined set of categories to an image [9]. Traditional approaches perform feature extraction and classification separately, while those based on DNNs integrate these steps into a single algorithm. DNN-based image classifiers, such as Convolutional Neural Networks (CNNs) and Vision Transformers (ViTs), have demonstrated outstanding performance in terms of Accuracy (ACC) compared with traditional image classification techniques. Despite their success, supervised learning methods require large quantities of labeled training data. While unlabeled data are often abundant and accessible, obtaining labeled datasets can be expensive in specific domains. Labeling may require domain experts, significant time investment, or involve complex multi-step procedures before annotation [10,11].

A particular approach to address this problem is ALrn, which aims to reduce both the number of labeled instances needed to achieve a given performance and the associated manual annotation by iteratively selecting the most informative unlabeled examples [10,12,13]. ALrn evaluates unlabeled data through a query strategy to measure its informativeness. These query strategies are divided mainly into two categories: information based and representation based. The former selects those samples that are more uncertain for the model; common uncertainty-based strategies are least confident, margin sampling, and query-by-committee. The latter attempts to represent the entire input space, often involving distance calculations, as seen in density-based or diversity-based strategies [10,12,13].

In ALrn, each iteration selects a set of images for annotation, a step whose computational cost increases with the size of the unlabeled set. In practical scenarios, the size of the unlabeled pool can be significantly larger than the labeled dataset, making this stage particularly critical from a scalability perspective [10]. The batch size determines how many samples are chosen at each iteration. Once these selected images are labeled, the model is retrained. In addition, the data selection process relies on sorting utility scores computed by the query strategy, a step whose computational cost increases with the size of the unlabeled set, which is typically large in DL applications. This iterative character, combined with the need to manipulate large data volumes, makes ALrn algorithms computationally expensive, especially in image classification due to the high dimensionality and complexity of the visual data [14]. In embedded applications, this problem is exacerbated by hardware and power constraints, as devices must perform training and data selection processes with limited computational and memory resources. These conditions hinder the practical implementation of ALrn algorithms in low-power environments, such as image sensor-based systems or intelligent edge platforms, where efficiency and operational autonomy are required [15,16].

Traditional computer platforms are based on the von Neumann architecture, where operations are executed sequentially by an Arithmetic Logic Unit (ALU) [17,18]. To reduce execution time, GPUs are widely used in Artificial Intelligence (AI) applications due to their coarse-grained parallelism, where multiple cores execute the same instruction simultaneously [17]. However, GPUs are optimized for high throughput rather than fine-grained control or custom dataflow. In this context, FPGAs offer customizable hardware capable of implementing fine-grained parallelism and pipelined data flows tailored to specific algorithmic needs [17,19,20]. These characteristics have led to the growing use of FPGA programmable logic as an alternative to CPU/GPU platforms in AI/machine learning (ML) workloads, where hardware flexibility enables customized processing cores that accelerate inference and reduce latency [21,22]. In addition to performance, energy efficiency is critical in embedded/edge environments. CPU/GPU platforms tend to have high power consumption, while FPGAs enable fine-grained parallelism, dataflow control, and techniques such as block shutdown and clock/frequency scaling, accelerating classification while maintaining lower power consumption than general-purpose solutions [16,23,24,25,26]. Despite this growing adoption, the iterative retraining and inference steps, as well as the required utility-score computation, make ALrn computationally demanding; specialized hardware acceleration for ALrn remains largely underexplored. In image classification, ALrn addresses the labeling problem by reducing the number of manually annotated samples, selecting only those images that provide the most information to improve the model. This significantly reduces the labeling effort without compromising the final performance. This feature is naturally complemented by FPGA-based architectures, as selecting more informative samples requires frequent, low-latency calculation of uncertainty metrics, which can be efficiently executed in parallel within the hardware. The combination of both strategies, therefore, reduces labeling costs while maintaining a fast decision-making flow suitable for embedded systems. This gap motivates the work presented in this paper. In particular, the instance selection stage, which involves computing uncertainty scores and performing top-*k* selection, has not been sufficiently explored from a hardware acceleration perspective, despite its impact on scalability.

While recent hardware-accelerated approaches have mainly focused on optimizing neural network inference or general top-*k* operations on FPGAs [25,26,27,28,29], the computational cost of the iterative instance selection step in ALrn has received comparatively little attention. To address this gap, we propose a hardware architecture that efficiently computes uncertainty scores and iteratively selects the top-*k* most informative samples. In this paper, we present the design and evaluation of a digital circuit for accelerating the instance selection stage of uncertainty-based ALrn using a query strategy. The proposed digital architecture computes utility values and performs in-line top-*k* selection without affecting the final performance of ALrn algorithms, returning the indices of the most informative samples. It is important to note that this work focuses on accelerating the query strategy stage, which becomes increasingly critical in large-scale scenarios where the size of the unlabeled pool significantly impacts the computational cost. The system is implemented and validated on a Xilinx 7000 SoC, combining an embedded ARM processor with programmable logic. The PYNQ framework is used to manage communication between the processor and the hardware accelerator, which is described in Verilog. Operating at 110 MHz, our implementation achieves a speedup of over two orders of magnitude compared to CPU- and GPU-based baselines, while consuming less than 2 W of power. The main contributions of our work are:

- A comparative analysis of uncertainty-based ALrn query strategies for image classification tasks.
- A novel in-line top-*k* selection algorithm tailored for hardware implementation.
- The design, implementation, and validation of a digital architecture for accelerating the instance selection stage using a query strategy in ALrn.

The rest of this paper is organized as follows. Section 2 reviews related work on ALrn and hardware acceleration. Section 3 presents the proposed workflow, integrating software and hardware stages, describes the datasets and pre-processing, details the ALrn algorithms employed, and outlines the proposed accelerator architecture. Section 4 reports validation, performance, and resource utilization results. Finally, Section 5 concludes the work and outlines future research directions.

## 2. Related Work

### 2.1. Active Learning Query Strategies for Computer Vision

The increasing availability of data and the success of machine learning models have motivated the use of ALrn algorithms to minimize labeling costs by iteratively selecting the most informative samples to improve the training dataset. ALrn has been applied across a range of computer vision tasks, including image segmentation [30,31,32], object detection [33,34], and image classification [35,36,37,38].

In image classification, many approaches rely on uncertainty-based query strategies. For example, Abdelwahab, Afifi, and Salama [39] combined least confident, margin sampling, and entropy uncertainty sampling with pseudo-labeling of confident instances, where samples with entropy below a predefined threshold are automatically labeled. This semi-supervised method outperformed traditional approaches on the Caltech-256 image dataset [40], achieving an ACC of 99.3%—a 25% improvement over the 74.3% obtained with conventional methods. Similarly, Gashi, Deng, and Elezi [41] benchmarked several selection strategies for ALrn, including random sampling, entropy, variation ratio, Bayesian ALrn, core-set, and learning-loss, across the classification datasets CIFAR-10 [42], CIFAR-100, Caltech-101 [43], and Caltech-256, showing that entropy outperformed other methods while maintaining algorithmic simplicity. Since uncertainty-based query strategies can select redundant data near decision boundaries, hybrid strategies that combine uncertainty and diversity have also been proposed. For instance, Sheng et al. [44] introduced the Two-Stage Batch-Mode ALrn (TBAL), where uncertain samples are first identified using the variation ratio, and then the least similar samples to the already labeled ones are selected from multiple clusters constructed using the k-means algorithm on the feature vectors of the most uncertain images. The TBAL framework demonstrated superior ACC compared with random-only, uncertainty-only, and diversity-only baselines.

### 2.2. Hardware Acceleration for Deep Learning Inference

Despite their benefits, ALrn algorithms are computationally intensive due to their iterative nature and the large volumes of data they handle. Just as in machine learning, ALrn is commonly implemented on CPU/GPU platforms [45,46,47], with very little attention given to specialized hardware acceleration for ALrn. At the same time, hardware acceleration for machine learning workloads, particularly for inference, has been extensively studied, with custom architectures achieving reductions in latency [21,22]. For example, Khaki and Choi [21] implemented VGG16 and VGG19 [48] on an Avnet Ultra96-V2 board using Vitis AI and TensorFlow 2. On the CIFAR-10 dataset, their FPGA-based implementation achieved top-1 accuracies of 89.54% for VGG16 and 87.46% for VGG19, which are nearly identical to the CPU baselines of 89.69% and 88.04%, respectively. The hardware design reduced the inference time from 4.75 to 0.65 ms/frame for VGG16 and from 5.59 to 0.85 ms/frame for VGG19, yielding speedups of 7.3× and 6.57×, respectively. Likewise, Lu et al. [22] proposed a heterogeneous accelerator for MobileNetV2 [49] on the PYNQ-Z2 board using Vivado for synthesis. In their architecture, the Processing System (PS) handled control and lightweight tasks, while the programmable logic accelerated convolutional and pooling layers for driver behavior recognition on the StateFarm dataset [50]. Their system achieved an inference time of 84.1 ms/frame, a 21.2× reduction relative to the ARM-only baseline, with ACC differing by less than one percentage point. In addition, the increasing complexity of machine learning algorithms and their growing demand in various applications have led to the development of open-source frameworks that facilitate the prototyping of these models on FPGAs, such as HLS4ML and FINN [51,52,53].

### 2.3. Specialized FPGA Architectures for Data Selection

Since most query strategies compute a utility value and then select the top-*k* or bottom-*k* samples, traditional implementations rely on sorting algorithms (e.g., *argmax* or *argmin*), which become a critical component in ALrn pipelines. While numerous FPGA-based sorting architectures have been proposed [54,55,56], these works primarily address general-purpose sorting rather than ALrn-specific implementations. A distinct line of research explores ALrn in FPGA contexts but with different goals. For instance, Ji et al. [57] addressed the challenge of predicting the inference latency of neural network architectures on FPGA devices. Their approach utilizes ALrn to iteratively select the most informative architectures for evaluation on hardware. This strategy reduces the latency collection cost by about 94% while maintaining nearly the same predictive ACC as a fully trained model. In a related line of research, ALrn has also been employed to guide the selection of representative hardware configurations, enabling exploration of the design space and accurate performance prediction with fewer evaluations [58,59].

Complementary to these approaches, several FPGA-based designs have specifically addressed top-*k* selection and partial sorting to reduce computational overhead. For example, streaming-based architectures enable single-pass processing of input data using pipelined and systolic structures, achieving high throughput without requiring full data reordering [28]. Other designs employ hybrid pipelined sorting architectures that combine bitonic networks with cascaded processing stages to extract the k-largest elements from continuous data streams efficiently [60]. More recent proposals integrate hybrid sorting strategies, such as quicksort and heap-based methods, to balance latency, throughput, and hardware resource utilization in top-*k* selection tasks [54]. These approaches highlight that efficient top-*k* selection in hardware depends on avoiding full data ordering, particularly in high-throughput scenarios and large data streams.

### 2.4. Limitations of Conventional Data Sorting in Active Learning

To the best of our knowledge, previous studies have focused mainly on accelerating inference stages or neural network components on hardware platforms such as FPGA, without addressing the specific computational bottlenecks of ALrn. Existing works emphasize the optimization of convolutional operations, normalization layers, and general top-*k* computations in streaming or vision tasks, yet none have explored hardware architectures explicitly designed to accelerate the query strategy that drives sample selection in ALrn [25,26,27,28,29]. For example, Aydin and Bilge [25] implemented an FPGA-based CNN accelerator for image registration, focusing on convolutional processing rather than sample-selection mechanisms. Similarly, Ebrahim and Khalifat proposed FPGA-optimized algorithms for identifying top-*k* elements in data streams, demonstrating efficient ordering strategies but without integrating them into ALrn cycles [28]. Other work, such as that by Xu et al., proposed a reconfigurable CNN architecture accelerated on an FPGA to improve the performance of convolutional operations [26]. Likewise, Kim et al. presented a hardware accelerator for approximate softmax and layer normalization in Transformer-type models, focusing on optimizing internal network components [29]. These works emphasize hardware-efficient DL computation but none explore architectures explicitly designed to accelerate the query strategy that drives sample selection in ALrn, highlighting the novelty of our work.

Finally, in the context of sorting, conventional sorting algorithms such as QuickSort and MergeSort have been implemented on FPGAs for ranking. Recent studies, such as those by Ben Jmaa et al. [61,62], evaluated these algorithms on Xilinx Zynq boards, showing how execution time increases significantly as the data volume grows. However, these implementations are designed for general-purpose sorting and do not consider the specific requirements of ALrn. Our work addresses this by integrating the margin metric calculation directly into a streaming selection architecture. Unlike conventional FPGA-based sorting approaches, the proposed architecture performs in-line top-*k* selection in a streaming fashion, enabling efficient processing as the unlabeled pool grows. Table 1 summarizes representative hardware-accelerated approaches and highlights the gap addressed in this work.

## 3. Materials and Methods

**Figure 1:**
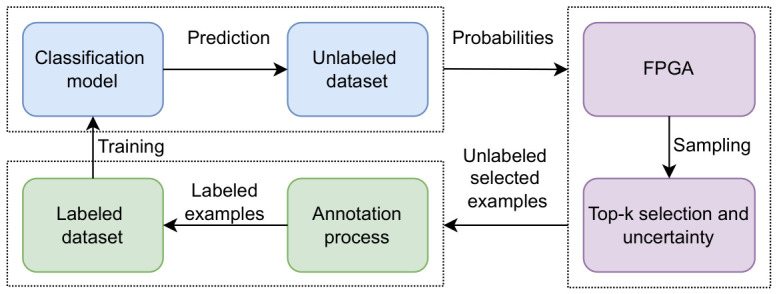
Conceptual diagram of the proposed FPGA-accelerated ALrn flow, showing the evaluation of the unlabeled set, the margin calculation, and top-*k* selection in hardware, as well as the incorporation of new labels into the training set. We accelerate the first iteration of ALrn, corresponding to the critical case where the unlabeled pool is at its maximum size.

Figure 1 presents a conceptual view of the complete flow of the proposed method. It illustrates how the classification model evaluates the unlabeled set to obtain predicted probabilities, which are then processed by the FPGA module to calculate the margin of uncertainty and execute top-*k* selection in an accelerated manner. The selected examples are then sent to the manual annotation process, and the new labels are incorporated into the training set, closing the iterative cycle of ALrn. In addition, it should be noted that the architecture was validated only in the critical case, corresponding to the first iteration, where the unlabeled set reaches its maximum size.

**Figure 2:**
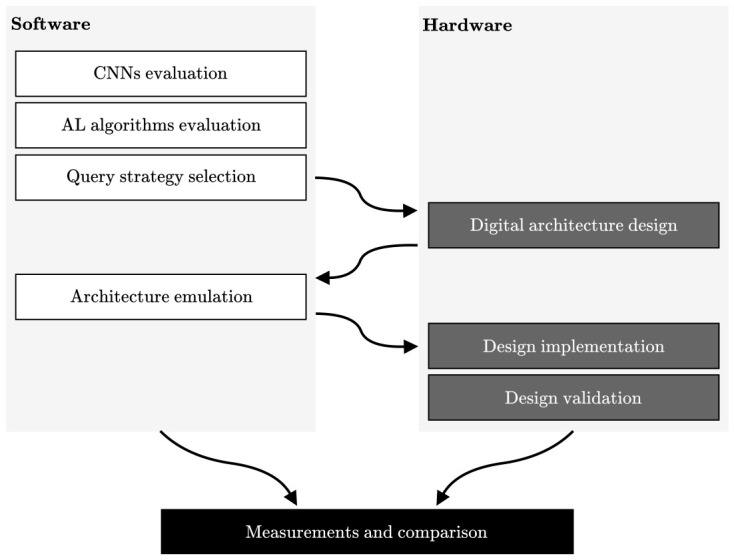
Overall workflow conducted, integrating software and hardware stages. The software side encompasses the evaluation of machine learning models, ALrn assessment, query strategy selection, and architecture emulation, while the hardware side involves digital architecture design, FPGA implementation, and validation. The workflow converges with a comparative analysis between software and hardware implementations.

On the other hand, Figure 2 outlines the workflow, spanning from initial evaluations to validation of the implemented hardware. On the software side, the employed CNNs are first evaluated on the image datasets, followed by the assessment of ALrn algorithms, where multiple uncertainty-based query strategies are compared in a pool-based setting. Based on this evaluation, the query strategy that offers the best results in terms of classification metrics is selected for hardware acceleration. The hardware flow begins with the design of a dedicated digital architecture for the selected query strategy. To support this process, a software-based emulation of the architecture’s functionality is conducted using Python 3.12, enabling the early exploration of design choices and their impact. Then, the design is implemented on the FPGA and validated. Finally, the workflow converges by comparing the hardware implementation against a traditional software implementation. For evaluation purposes, the Xilinx Vivado software is used to estimate the resource utilization and power consumption of the design.

### 3.1. Datasets and Pre-Processing

We evaluated the performance of ALrn algorithms on four datasets: Fashion-MNIST and CIFAR-10, which are widely used standard benchmarks, and Chest X-Ray and DeepWeeds, which represent more realistic, real-world conditions. Fashion-MNIST consists of grayscale images of clothing items, while CIFAR-10 comprises color images of animals and vehicles [42,63]. On the other hand, the Chest X-Ray dataset contains grayscale radiographic images in RGB format, while DeepWeeds consists of in situ color images of multiple weed species captured in natural agricultural environments and characterized by background clutter [5,6]. Thus, the selected combination of benchmark and application-oriented datasets enables a comprehensive evaluation of the proposed ALrn strategies under both controlled experimental settings and real-world conditions [64,65,66,67]. Table 2 summarizes the main characteristics of all datasets, including resolution, images per class, and categories. All images were resized to 224 × 224 pixels to comply with the input requirements of standard CNN architectures, such as MobileNet, EfficientNet, and ResNet50. For Fashion-MNIST, the single grayscale channel was replicated to form 3-channel inputs. Model-specific pre-processing was applied in all cases.

It is important to highlight that the proposed FPGA accelerator is resolution agnostic, as it operates on class probability vectors and scales with the number of classes and the batch size used for sample selection.

### 3.2. Classification Models

Three CNN architectures are used: MobileNetV1, EfficientNetB0, and ResNet50 [68,69,70]. In particular, MobileNetV1 and EfficientNetB0 are selected for their relatively low parameter counts. In contrast, ResNet50 is included to assess the behavior of ALrn when applied to a model with more parameters. All models are used as feature extractors, pre-trained on the ImageNet ILSVRC dataset [71], with an output layer added that contains as many neurons as there are classes in the target datasets. Table 3 shows the total number of parameters and the number of trainable parameters for each architecture.

Model-specific pre-processing is applied according to the architecture utilized as follows [68,69,70]:

- **MobileNetV1:** input pixels values are scaled to the range [−1,1].
- **EfficientNetB0:** no additional pre-processing is applied (pass-through).
- **ResNet50:** input images are converted from red, green and blue (RGB) to blue, green and red (BGR) and each color channel is zero-centered with respect to the ImageNet dataset, without scaling.

To evaluate classifier performance, we use two standard metrics for image classification: ACC and F1-score (F1), defined mathematically as

(1) $$\mathrm{ACC}=\frac{TP+TN}{TP+TN+FP+FN},$$

(2) $$F1=\frac{2TP}{2TP+FP+FN},$$

where TP, TN, FP, and FN correspond to classification results: true positive, true negative, false positive, and false negative, respectively [9,35,36].

Table 4 summarizes the training hyperparameters used in all experiments. We employ 5-fold cross-validation; therefore, the results correspond to the mean across the five folds. Across all datasets, a randomly selected, balanced subset of 6400 images is used, with an 80/20 train–test split applied within each fold. This design choice simplifies the number of learning-curve iterations, given the batch size of 512.

Table 5 presents the classification performance of MobileNetV1, EfficientNetB0, and ResNet50 on the Fashion-MNIST, CIFAR-10, Chest X-Ray, and DeepWeeds datasets. It can be observed that, in most cases, EfficientNetB0 achieves the best performance across the datasets in terms of ACC and F1, outperforming MobileNetV1 and ResNet50.

### 3.3. Active Learning Algorithms

**Figure 3:**
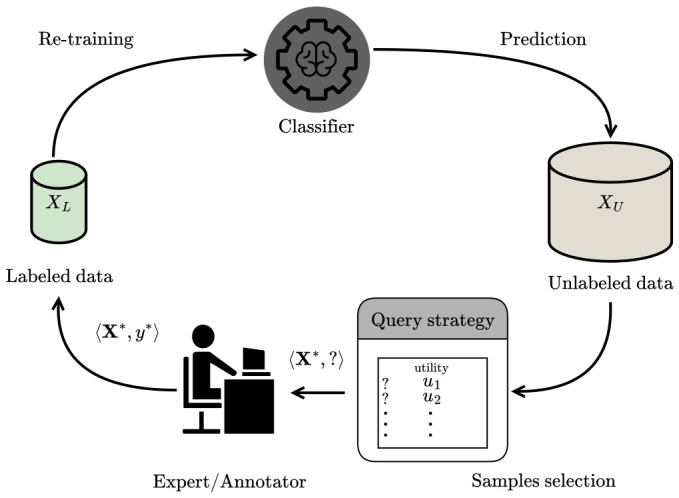
Schematic representation of the pool-based ALrn framework. An initially small labeled dataset is used to train a classifier. The classifier, in conjunction with a query strategy, evaluates the large unlabeled data pool to estimate informativeness and select relevant samples. Selected samples are then sent to an expert annotator for labeling and subsequently added to the labeled pool, thereby improving the classifier iteratively.

As illustrated in Figure 3, we adopt a pool-based ALrn setting, where a small subset of labeled data is initially available, and the remaining data forms a large unlabeled pool. In addition, all model evaluations are performed using five-fold cross-validation, which provides more stable performance estimates and ensures consistent comparisons across the evaluated methods. Although the analysis focuses on the critical case, i.e., the first iteration of the ALrn cycle, this validation scheme provides a reliable estimate of the models’ overall performance, ultimately reporting the average across the five folds.

For each CNN, we evaluate three uncertainty-based query strategies: least confident, margin sampling, and entropy. Their performance results are compared both among themselves and against a passive learning (PLrn) baseline, in which instances are selected randomly. In each ALrn iteration, a batch size of 10% of the initial dataset is used, and the selected samples are then added to the labeled dataset.

**Figure 4:**
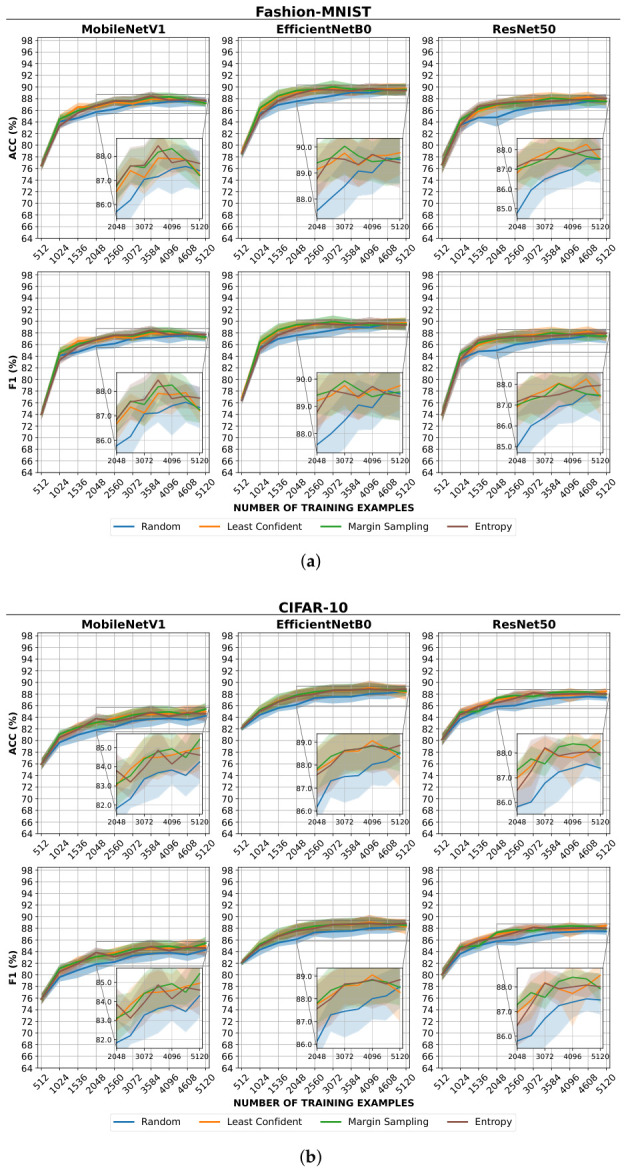
Learning curves of Active Learning strategies compared to passive learning for (**a**) Fashion-MNIST and (**b**). The shadowed bands represent the standard deviation estimated from 5-fold validation. In both datasets, active learners outperform the passive baseline for most quantities of labeled data. On Fashion-MNIST, margin and entropy sampling with MobileNetV1 exceed 88% accuracy and F1 using 70% of the training set, while EfficientNetB0 reaches above 89% with only 60% of labeled data. On CIFAR-10, active strategies achieve near-maximum performance with 40–60% fewer labels compared to passive learning. The inset in each subplot shows a magnified view of the learning curves over the final 60% of the training pool (2048–5120 samples).

**Figure 5:**
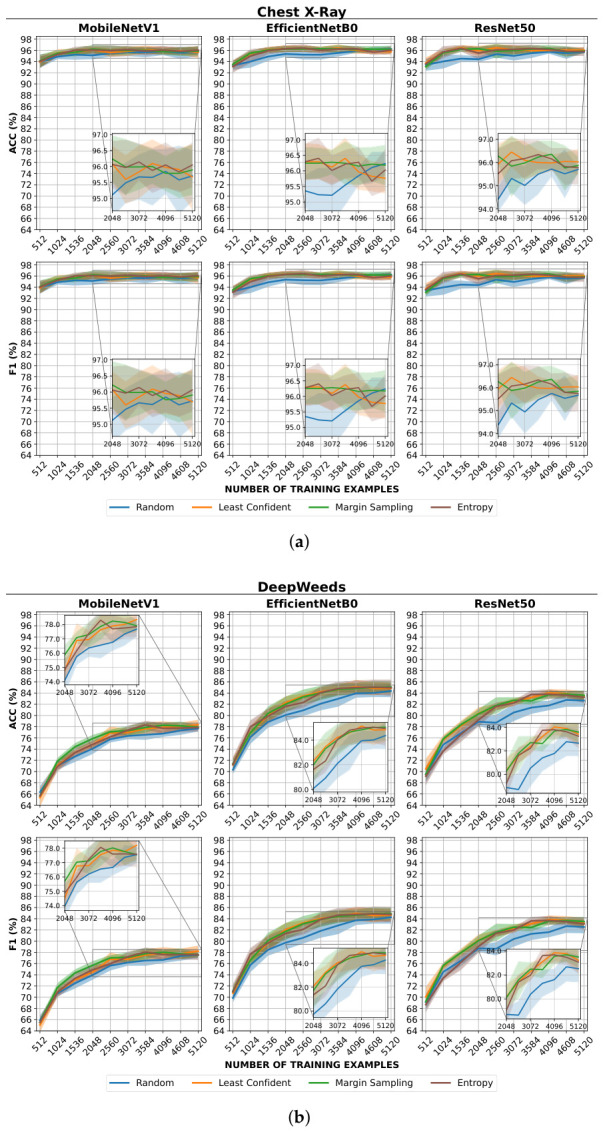
Learning curves of Active Learning strategies compared to passive learning for (**a**) Chest X-Ray and (**b**) DeepWeeds. The shadowed bands represent the standard deviation estimated from 5-fold validation. In both datasets, active learners outperform the passive baseline for most quantities of labeled data. For the Chest X-Ray dataset, all three models achieve accuracy and F1 scores above 95.5% using only 30% of the labeled data when employing Active Learning query strategies, differing by less than 1% from the performance obtained when training with the full dataset. A similar trend is observed for DeepWeeds, where Active Learning enables comparable performance using approximately 70–80% of the available training data. The inset in each subplot shows a magnified view of the learning curves over the final 60% of the training pool (2048–5120 samples).

After the training/testing split, 5120 examples are used for training, from which 512 (10%) are initially labeled to form the initial labeled set, and the remaining 4608 constitute the unlabeled pool. In each iteration, 512 images are selected according to the employed strategy. Figure 4 and Figure 5 show the learning curves of the classifiers according to the number of training examples and the performance obtained in terms of ACC (%) and F1 (%). Active learners achieve higher performance than the PLrn baseline for almost all training set sizes evaluated. In addition, a two-tailed Wilcoxon signed-rank test (α = 0.05) is applied to assess whether the differences in performance between margin sampling and the other ALrn query strategies are statistically significant. The input data for this test consists of the Accuracy and F1-score values at each iteration of the learning curves. The null hypothesis ($H_{0}$) states that there is no significant difference in performance between margin sampling and the alternative ALrn strategies, while the alternative hypothesis ($H_{1}$) assumes a significant difference. The results show that in all cases, no significant differences are found (i.e., $p_{\mathrm{value}}>\alpha$), except for three comparisons: MobileNet on DeepWeeds against least confident and entropy, and EfficientNet on Fashion-MNIST against entropy. This supports using margin sampling, as it offers competitive performance compared to more complex strategies such as entropy while incurring a considerably lower computational cost, which facilitates its implementation in hardware. A more detailed analysis of the performance of ALrn strategies is presented in Appendix A, which includes Table A1, Table A2, Table A3, Table A4, Table A5, Table A6, Table A7, Table A8, Table A9, Table A10, Table A11 and Table A12. These tables report, for each combination of model, dataset, and query strategy, the minimum number of labeled examples required to achieve different levels of accuracy and F1. This numerical breakdown complements Figure 4 and Figure 5 and allows for an accurate characterization of the comparative behavior of the strategies throughout the iterations of the ALrn process.

In Table 6, the Area Under the Learning Curve (AULC) values for all the learning curves shown above are presented, and it is computed as follows:

(3) $$\mathrm{AULC}=\frac{1}{2(n_{AL}-1)}\sum_{i=1}^{n_{AL}}\left(P1_{i-1}-P1_{i}\right),$$

where $n_{AL}$ is the number of points of the learning curve, and $P_{i}$ is the performance at iteration *i*. In most cases, margin sampling has the largest AULC, which further confirms its suitability for hardware acceleration.

### 3.4. Selection Algorithm

Mathematically, margin sampling is expressed as shown in Equation (4):

(4) $$x^{*}=\underset{x\in X_{U}}{argmin}\;P_{h}\left({\hat{y}}_{1}|x\right)-P_{h}\left({\hat{y}}_{2}|x\right),$$

where $x^{*}$ denotes the most uncertain instances, and ${\hat{y}}_{1}$ and ${\hat{y}}_{2}$ are the most probable and the second most probable labels, respectively, under the model *h*.

As shown, margin sampling relies on the argmin operation to obtain the indices of the samples with the smallest margins. Software approaches to this problem include sorting-based algorithms, which fully order the data at logarithmic or quadratic complexity, and selection-based algorithms, which retrieve the *k* smallest elements without complete ordering. Although selection algorithms can achieve linear average-case complexity, this is not guaranteed in the worst case. Regardless of the method employed, all margin values must be computed and stored in memory prior to selection, which limits feasibility in resource-constrained devices such as FPGAs, where on-chip memory is scarce. The proposed hardware architecture addresses this constraint by processing data in a streaming fashion, maintaining only a fixed set of candidate indices without requiring full storage of the margin array, and achieving deterministic linear complexity independent of input distribution.

Our first proposed approach avoids using a sorting algorithm by employing a memory unit whose size matches the batch size, i.e., the number of instances selected in each ALrn iteration. Algorithm 1 describes this selection method. Initially, the memory unit is filled with the first *k* margins received, and the maximum stored value is calculated. Subsequently, each incoming margin is compared to the maximum value, and if it is smaller, it replaces the maximum value in the memory unit; a new maximum value must then be found. At the end of the process, the memory unit contains the *k* smallest margins in the input stream.
**Algorithm 1:** Selection of indices using one memory unit.
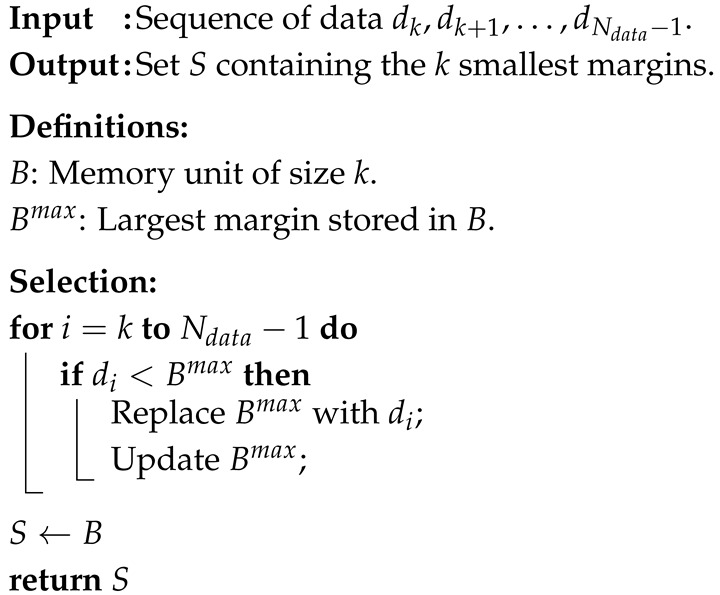


Most image classifier accelerators in the literature operate in a streaming or pipelined fashion, typically reporting throughput in terms of frames per second. To remain consistent with these works, we assume that classifier results arrive at a rate of one per clock cycle. Under this assumption, a limitation of Algorithm 1 arises since the maximum value stored must be calculated within a single clock to allow timely comparison with the incoming margin, which is not possible in the current context. To address this limitation, we propose an alternative approach described in Algorithm 2. In this method, multiple memory units of equal size are employed. During the initial phase, all memory units are filled sequentially with incoming margins. Thereafter, the next incoming margin is compared with the maximum value stored in the first memory. If it is smaller, the memory unit is updated, and a new maximum value calculation is triggered. The next incoming margin is compared with the second maximum value in memory, and this process continues. Therefore, the number and size of the memory units are chosen to ensure that by the time a given memory unit is revisited for comparison, its maximum value has already been calculated. This method enables in-line data selection with reduced memory requirements compared to traditional sorting-based approaches. The correctness of this method relies on the fact that each memory bank processes a disjoint subset of the margin values and retains the smallest local candidates within its assigned range. The global candidate set is obtained by merging all local candidates, and the final top-*k* selection is performed over this merged set. Since each index appears in exactly one bank and all local minima are included in the global stage, the method guarantees that the *k* smallest margins of the entire dataset are recovered, even without sorting all the data. As each memory unit produces its own maximum value, the proposed algorithm does not strictly select the smallest margins. To evaluate the impact of Algorithm 2 within the ALrn pipeline, we compare the indices selected by this method against those obtained using a baseline sorting-based implementation.
**Algorithm 2:** Selection of indices using multiple memory units.
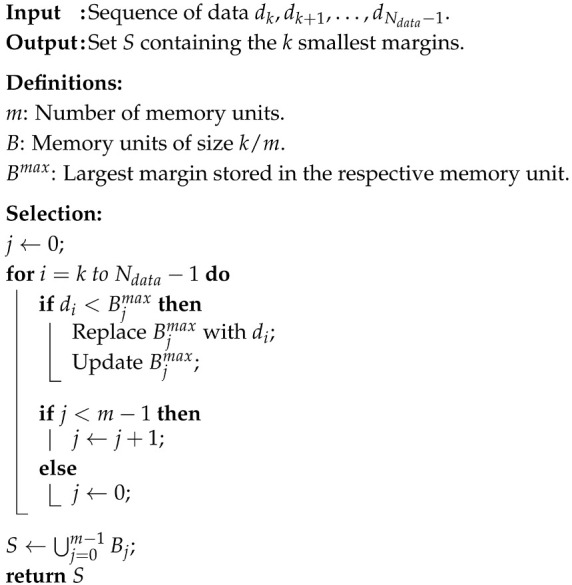


**Figure 6:**
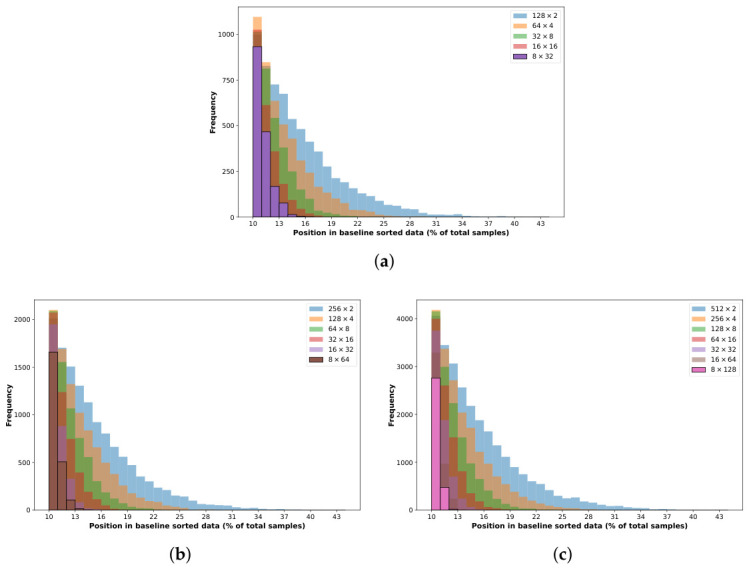
Distributions of mismatched indices between the proposed selection algorithm and the baseline sorting-based implementation across different memory configurations. (**a**–**c**) show results for batch sizes of 256, 512, and 1024, respectively. Labels indicate the number of memory units and their corresponding size (units/size). Error refers to the position of the mismatched indices within baseline’s sorted total data. For example, with a batch size of 256 and a 128×2 configuration, mismatched indices lie mostly between 10 and 35%, resulting in an error of approximately 15%, whereas with an 8×32 configuration, the error decreases to around 3%.

Our first test analyzed the proposed algorithm’s performance in comparison to the baseline sorting method. Specifically, we examined the indices selected by the proposed algorithm that were not included among the baseline’s selected initial images. For these mismatched indices, we determined their positions within the baseline’s complete sorted data to quantify how far they deviate from the true top-*k* samples. The farther these mismatched indices are positioned from the 10% in the baseline sorted data, the larger the selection error. Figure 6 shows the distribution of these mismatched index positions, while Table 7 reports the number of mismatched indices observed across different memory configurations. For each configuration, randomly generated balanced data were permuted 100 times, and the table presents the mean values of the minimum, maximum, average, and standard deviation of the mismatched index counts across these 100 runs. Fewer mismatched indices indicate better alignment between the proposed algorithm and the baseline selection. As shown in Table 7, the number of mismatched indices decreases as the number of memory units increases and their size decreases, indicating better alignment with the baseline selection in terms of raw count. However, as observed in Figure 6, for a given batch size, configurations with fewer memory units of larger size produce a more concentrated distribution of mismatched index positions, closer to the 10% boundary of the baseline sorted data, indicating that the mismatched indices are near-boundary cases that would have a marginal impact on ALrn performance. These two observations are complementary: while more units reduce the total number of mismatches, fewer units of larger size place the remaining mismatches closer to the selection boundary, resulting in a better approximation in terms of practical impact on the selected pool.

The choice of memory configuration involves a trade-off between approximation quality and pipeline feasibility. For a fixed batch size, increasing the number of memory units improves raw mismatch count, while increasing their size improves the distribution quality of mismatched indices. However, larger memory units require more cycles to compute their maximum value, imposing a feasibility constraint: the number of units must be at least $\log_{2}\left(N\right)$, where *N* is the unit size, to ensure the maximum value is ready before each unit is revisited. For a batch size of 512, the configuration that maximizes unit size while satisfying this constraint is 8 units of size 64, which prioritizes distribution quality, placing mismatched indices closest to the 10% selection boundary over raw mismatch count reduction.

**Figure 7:**
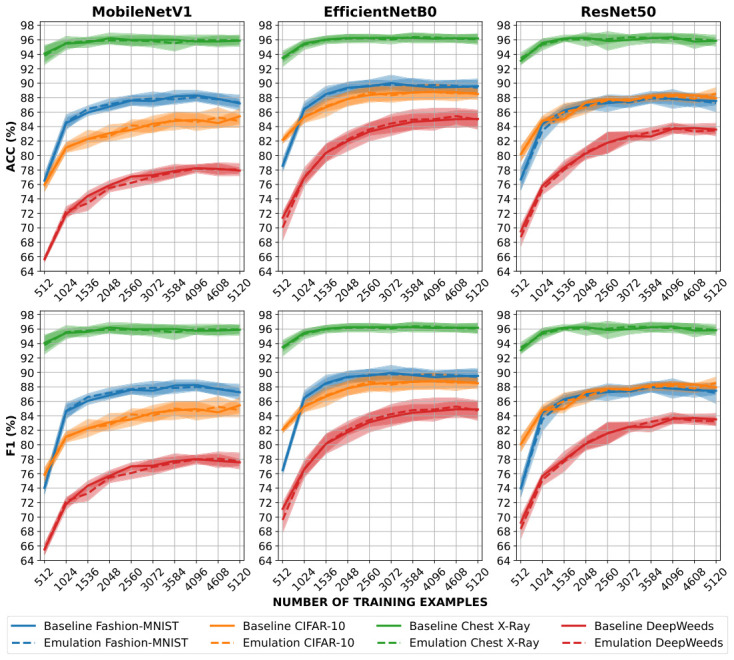
Learning curves of the classifiers using margin sampling, comparing the baseline sorting-based selection method with the proposed multiple memories approach across multiple datasets and network architectures. The shadowed bands represent the standard deviation estimated from 5-fold validation. The largest appreciable difference occurs for ResNet50 on CIFAR-10 with 1536 training examples, where the mismatch reaches approximately 1% in both ACC and F1 metrics.

Our second test, shown in Figure 7, compares the learning curves obtained using the baseline sorting-based selection method and a software emulation of the multiple memory approach for a batch size of 512. The resulting curves are almost identical in terms of performance metrics. Furthermore, as presented in Table 8, the most significant observed difference in AULC between the two methods is 0.0023.

### 3.5. Accelerator Architecture

**Figure 8:**
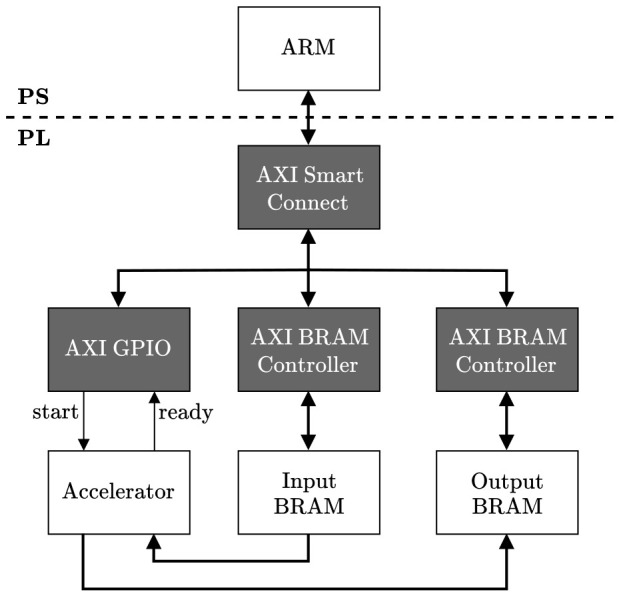
Proposed system architecture. The PS interfaces with the accelerator through an Advanced eXtensible Interface (AXI) SmartConnect module, which manages the interconnection between the PS, AXI BRAM Controllers, and AXI General-Purpose Input/Output (GPIO). The two AXI BRAM Controllers handle input and output data storage, while the AXI GPIO provides start and ready signals to coordinate processing.

Figure 8 illustrates the general system architecture for accelerating the margin sampling query strategy within ALrn. The SoC comprises two main components: the PS, which contains the ARM processor, and the programmable logic (PL), which integrates the reconfigurable hardware. The predicted probabilities are initially stored in a Double Data Rate (DDR) memory, which hosts the Linux file system. The PYNQ framework facilitates the software–hardware interaction by providing Python-based access to the memory-mapped AXI peripherals. Using the PYNQ MMIO class, the PS maps the physical addresses of the AXI BRAM controllers and AXI GPIO to the Python environment, enabling Direct Memory Access and control signal management. Data is transferred from the DDR to the input Block Random Access Memory (BRAM) via the ARM processor through sequential write operations to the mapped BRAM address space. The accelerator reads input data from the BRAM, performs the required computations, and writes the results to the output BRAM. Processing is initiated by writing to the AXI GPIO start signal register, and completion is detected by polling the ready signal register. Once the ready signal is asserted, the PS retrieves the resulting indices by reading from the output BRAM mapped address space.

It is worth noting that this interface relies on sequential MMIO transactions issued through the ARM processor, where each 32-bit write or read operation is handled individually without bursting. For an input of 4608 samples, corresponding to the critical case defined below, each represented as a 256-bit word requiring eight 32-bit transactions, the input transfer alone involves 36,864 sequential write operations. This sequential, non-burst nature of the transfer is inherent to the MMIO-based prototyping interface and does not reflect the throughput achievable with an optimized PS/PL communication scheme such as AXI Direct Memory Access (DMA).

The margin sampling query strategy implemented by the accelerator can be divided into two stages:

1. **Margin computation stage**: Computation of the margin (difference between the two highest probabilities.)
2. **Data selection stage**: Comparison, storage, and selection of the smallest margins using multiple memories algorithm (Algorithm 2).

**Figure 9:**
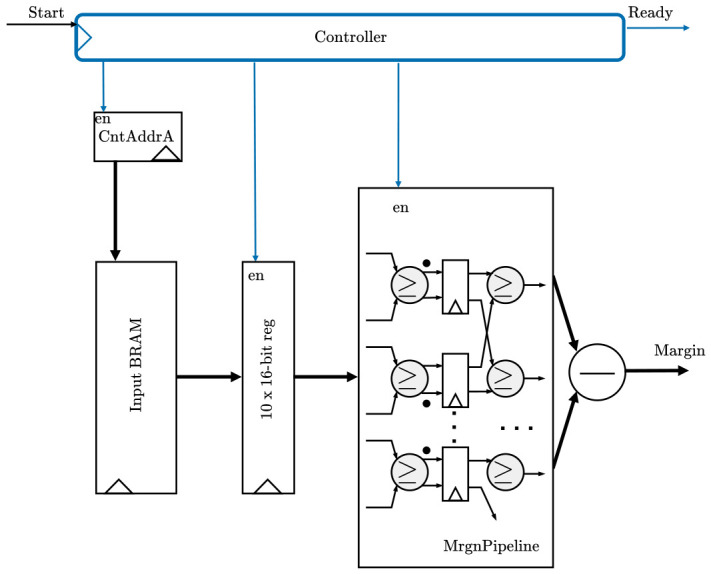
Hardware architecture for margin computation stage of the accelerator: margin calculation. Predicted probability vectors are read sequentially from the input BRAM via a dedicated address counter (*CntAddrA*) and stored in a 10 × 16-bit register array. Each 256-bit BRAM word contains 160 bits representing 10 class probabilities encoded in 16-bit fixed-point format. This stage prepares the data for the subsequent pipelined comparator tree, which identifies the two highest probabilities per instance.

Figure 9 shows the hardware architecture for margin calculation. Each vector of predicted probabilities is stored in the input BRAM as a 256-bit word, with 160 bits representing the probabilities encoded in 16-bit fixed-point format. A dedicated counter (*CntAddrA*) generates a new BRAM address in each clock cycle, enabling sequential reading of the input data. The retrieved probabilities are stored in a 10 × 16-bit register array.

**Figure 10:**
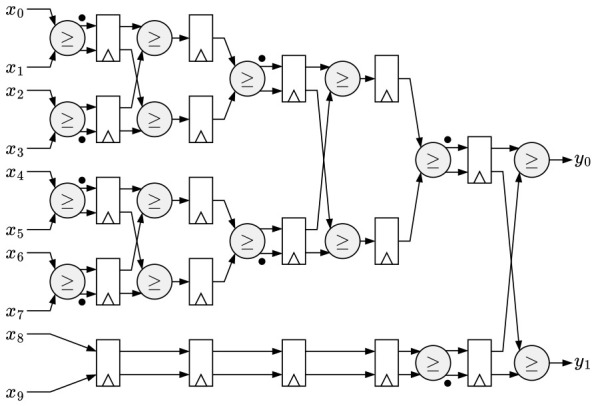
Pipelined comparator tree (*MrgnPipeline*) used in the margin computation stage to identify the two highest predicted probabilities from each input instance. The black dots adjacent to each comparator indicate the larger value resulting from the comparison. Comparisons are organized into groups of four inputs. Initially, pairwise comparisons are performed, followed by cross comparisons between the larger value of one pair and the smaller value of the other to identify the top two values. When a complete group of four inputs is not available, the inputs are registered until a group of four can be formed.

Figure 10 illustrates the pipelined comparator tree (*MrgnPipeline*) employed in the margin computation stage. This module leverages both spatial and temporal parallelism to identify the two highest probabilities within $\log_{2}\left({N_{classes}}^{2}\right)$ clock cycles, where $N_{classes}$ is the number of classes in the dataset. The inputs to the comparator tree are the predicted probabilities of a single instance read from the register array, and the outputs are the two most significant probability values identified. The final subtractor computes the margin as the difference between these two highest probabilities. This data flow from input BRAM to margin calculation is analogous to the *for* loop iteration over the margins in Algorithm 2.

**Figure 11:**
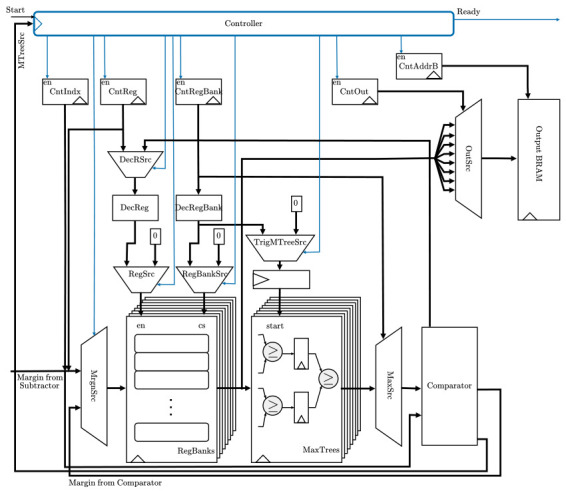
Data selection stage hardware architecture for margin comparison and selection. Computed margins are concatenated with their indices and register identifiers before storage in register banks (*RegBanks*). Multiplexers control input selection during initialization and replacement, while counters and decoders address specific registers. Pipelined comparator trees (*MaxTrees*) identify maximum values within each bank, and a comparator module (*Cmp*) determines replacements. A central controller manages all select signals and operations to coordinate data flow.

Figure 11 shows the hardware employed in the data selection stage. The memory units of Algorithm 2 are register banks (*RegBanks*) that enable the simultaneous reading of all stored values for maximum-value calculation. Before storage, each computed margin is concatenated with its corresponding index and register identifier for replacement purposes, both generated by dedicated counters (*CntIndx* and *CntReg*). A multiplexer (*MarginSrc*) is placed at the inputs of the register banks. While the memory units are being initialized (filled), the multiplexer selects the concatenated output from the subtractor module. Once initialization is complete, it switches to selecting the output from the comparator module (*Cmp*). Register banks and specific registers within them are addressed using dedicated counters and decoders (*CntReg*, *CntRegBank*, *DecReg*, and *DecRegBank*). The multiplexers at the inputs of the register banks (*RegSrc* and *RegBankSrc*) have a zero input to avoid writing any value when a replacement is not necessary (i.e., when the incoming margin is not smaller than the maximum value stored in the memory unit).

The stored values of the register banks are connected to the maximum calculation modules, which determine the most significant margin within each bank using pipelined comparator trees (*MaxTrees*), and to an output multiplexer (*OutSrc*) that selects the appropriate index to be written into the output BRAM at the end of processing. The maximum values computed by comparator trees are fed to a multiplexer (*MaxSrc*) at the comparator input, which selects the corresponding maximum value to be compared against the incoming margins. The comparator returns the minimum value, along with its corresponding register address for storage. The decoder used for register selection (*DecReg*) includes a multiplexer (*DecRegSrc*) at its input, enabling it to decode register addresses from the comparator once the memory units are filled, following the initial processing phase. All select signals for multiplexers, as well as counters, enable, and maximum trees start signals, are managed by a controller module that orchestrates the overall data flow and processing sequence within the accelerator.

**Figure 12:**
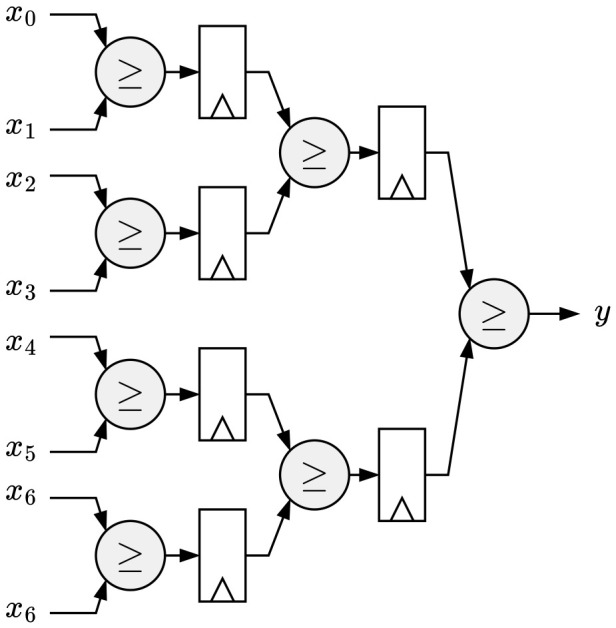
Simplified three-stage pipelined comparator tree representative of the structures used in each register bank for maximum value calculation. Inputs are all stored margins within a bank, and the output is the largest margin identified. The number of stages grows logarithmically with bank size, achieving a latency of $\log_{2}\left(N\right)$ clock cycles, where *N* is the register bank size.

Figure 12 presents a simplified three-stage pipelined comparator tree, representative of those used in the design (*MaxTrees*) for calculating the maximum value within each bank. The inputs for a particular tree are all the corresponding margins from a bank, and the output is the most significant margin identified among them. The number of stages in the tree scales logarithmically with the size of the register bank, achieving a latency of $\log_{2}\left(N\right)$ clock cycles, where *N* is the size of the register bank.

**Figure 13:**
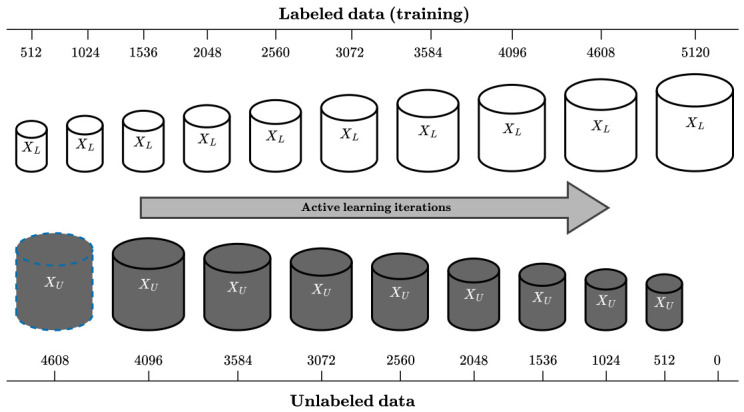
Evolution of labeled and unlabeled pools across ALrn iterations for a batch size of 512. The critical case, corresponding to the first iteration with the largest unlabeled pool, is shown as the blue dashed line (–).

Due to the limited resources available on the FPGA, the largest implementable batch size is 512. A batch size at the next power of 2 (i.e., 1024) is not feasible on this device. According to the results of Figure 6, the best feasible memory unit configuration is eight units of size 64. The architecture is designed for a critical case, where both the number of data points and the number of classes to be processed are known in advance. Figure 13 illustrates how the labeled and unlabeled data pools change over ALrn iterations for a batch size of 512. Considering this, the critical case is defined as the scenario where the query strategy is applied to the largest number of unlabeled instances, that is, in the first iteration of ALrn, when the unlabeled pool has 4608 instances. Therefore, each register in the register banks is large enough to store the 16 bits of the margin, 13 bits to represent the index (ranging from 0 to 5119), and 6 bits for the register address.

## 4. Results and Discussion

This section presents the results obtained from the complete system implementation and compares them against those from a traditional software platform. In addition, the performance analysis is contextualized with respect to the system-level execution time of a full ALrn iteration, including training, inference, and query strategy stages.

### 4.1. Validation

Two tests were conducted to validate the implemented design, both involving a comparison of the indices selected by the hardware accelerator with those obtained from a software emulation of Algorithm 2. The tests are described as follows:

1. **Random integer test**: In each of the 50 iterations, a new set of inputs was generated by creating 10 random integers per instance, in the range of $\left[0,2^{16}-1\right]$.
2. **Classifier predicted probabilities test**: Mismatch evaluation was performed using the predicted probabilities produced by the classifiers. We considered the Fashion-MNIST and CIFAR-10 datasets because their ten classes correspond to the most critical and computationally demanding cases within the datasets, due to their impact on processing time and, consequently, on the digital system design. This test was applied to a total of six sets of predicted probabilities, corresponding to the output probability vectors of each of the three classification models evaluated on both datasets. Each 32-bit floating-point probability value was multiplied by $2^{16}$ (<<16) to match the fixed-point representation format used in the hardware.

In both tests, no mismatched indices were observed between the hardware accelerator and the software emulation, confirming the functional correctness of the implemented design.

### 4.2. Performance and Resource Utilization

We synthesized the design and analyzed its performance using the Xilinx Vivado 2024.2 design suite. We implemented the proposed accelerator on a ZYNQ-7000 XC7Z020-1CLG400C SoC, which includes an ARM processor used in this work for communication tasks, and an Artix-7 FPGA where the architecture is implemented. On the other hand, the software implementation used for comparison was executed on the Google Colaboratory platform, which utilizes a 2.2 GHz Intel Xeon processor (Broadwell microarchitecture, CPU family 6, model 79) with 55 MB of cache memory, a physical core with two threads (hyperthreading enabled), and 13 GB of RAM. The baseline implementation used Python 3.12.12 with NumPy 2.0.2, which is configured with OpenBLAS 0.3.27 as the underlying BLAS library for optimized numerical operations, though the sorting algorithms themselves rely on NumPy’s native C-based implementations. Although the cloud platform does not report the specific Xeon model designation or the actual power consumption during execution, processors with these specifications, designed for servers and data centers, typically have a Thermal Design Power (TDP) in the range of 120 to 150 W [72].

Equation (5) defines the total number of clock cycles required by the hardware accelerator to complete processing:

(5) $$N_{cycles}=\log_{2}\left({N_{classes}}^{2}\right)+N_{data}+\left(N_{R}\times N_{RB}\right),$$

where $N_{classes}$ is the number of classes in the classification task, $N_{data}$ is the number of the input data instances, $N_{R}$ is the number of registers per register bank, and $N_{RB}$ is the total number of register banks. The first term represents the initial delay of the pipelined two-highest comparison tree, specifically computed as $\log_{2}\left({N_{classes}}^{2}\right)-1$ clock cycles. The product $\left(N_{R}\times N_{RB}\right)$ corresponds to the batch size and accounts for the number of cycles needed to write the resulting indices to the output BRAM.

For the implemented digital architecture, the parameter values are $N_{classes}=10$, $N_{data}=4608$ (critical case), $N_{R}=64$ and $N_{RB}=8$. This configuration results in a total of 5128 clock cycles for processing. Equation (5) characterizes the PL accelerator processing time exclusively, independent of the PS/PL data transfer overhead. Consequently, the reported execution time corresponds strictly to PL processing. Operating at a maximum clock frequency of 110 MHz, the accelerator achieves a PL processing time of 46.15 μs.

Table 9 summarizes the execution times measured across software baselines and the hardware accelerator for the critical case. Software measurements represent the mean of 1000 independent runs to ensure statistical reliability. Python-based implementations are included as the primary reference since Python is the dominant language in ML and ALrn workflows. C++ implementations, compiled with flags -O2 -std=c++17, are additionally reported to isolate the contribution of the hardware architecture from language-level performance differences. As shown, the hardware accelerator achieves a speedup of 231.7× over the traditional Python sorting-based baseline and 22.9× over the best software baseline, namely the proposed algorithm implemented in optimized C++. Note that the Python implementation of the proposed algorithm incurs higher execution time than Python-based sorting due to interpreter overhead in the selection logic, an inefficiency that is eliminated in the optimized C++ implementation. These speedups correspond specifically to the computation of the query strategy. As discussed previously, the overall impact on the ALrn pipeline depends on the relative contribution of this stage, which increases with the size of the unlabeled pool.

Consequently, the speedups of 231.7× and 22.9× reported in Table 9 correspond strictly to PL processing. To provide a complete end-to-end characterization as required for a fair system-level comparison, the total execution time including PS→PL transfer, PL processing, and PL→PS transfer was measured directly on the ARM processor using memory-mapped I/O from a C program, eliminating the Python interpreter overhead from the measurement. The end-to-end time decomposes as follows: the PS→PLrn input transfer contributes 14.838±0.768 ms, PLrn processing and done-signal polling contribute 0.051±0.007 ms, and the PL→PS output transfer contributes 0.210±0.027 ms, resulting in a total end-to-end time of 15.098±0.767 ms. As shown, the PS→PL input transfer dominates the end-to-end time, accounting for 98.3% of the total, while PL processing represents only 0.34%. This disproportion is inherent to the current Memory-Mapped Input–Output (MMIO)-based prototyping interface, which issues 36,864 sequential 32-bit transactions over the AXI General Purpose port to transfer 4608 probability vectors of 256 bits each, without burst capability. Consequently, the end-to-end time reflects the communication interface overhead rather than the computational efficiency of the hardware architecture. As discussed in the scalability section, replacing the current MMIO interface with AXI DMA would eliminate this bottleneck through burst transfers, directly improving the end-to-end execution time while preserving the linear complexity advantage of the PL accelerator.

**Figure 14:**
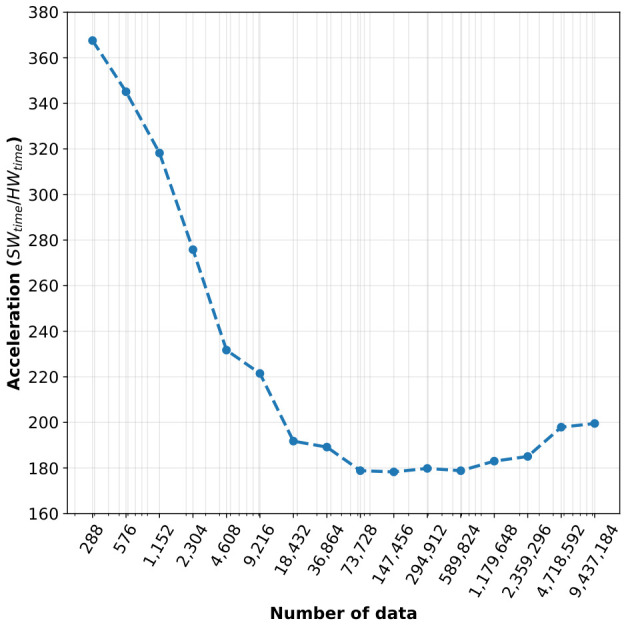
Estimated acceleration factor achieved by the hardware accelerator compared to the software baseline implementation, as a function of the total number of processed data instances. Hardware times are computed using Equation (5), while software times are measured using randomly generated input data with a batch size equal to 10% of the total data. As shown, acceleration is at least greater than 170×, with a linear increase in acceleration observed for dataset sizes above 589,824 instances.

Figure 14 shows the estimated acceleration curve, based on software measurements using randomly generated data (with a batch size of 10% of the total data) and hardware processing time estimated using Equation (5). The curve spans data sizes from 288 to 9,437,184 samples, reaching peak acceleration above 300× for the smallest evaluated sizes, stabilizing near 180× for datasets larger than 36,864 samples, and then increasing linearly with data size. This trend further supports the suitability of the proposed architecture for large-scale ALrn scenarios.

Our implementation runs at a maximum clock frequency of 110 MHz, whereas the ARM processor operates at 650 MHz. Increasing the programmable logic frequency beyond this point causes the accelerator to fail timing requirements, with critical paths located around the register banks. At the maximum clock frequency, Xilinx Vivado estimates the total on-chip power consumption to be 1.884 W, comprising 0.153 W of static power and 1.731 W of dynamic power. The ARM processor accounts for 1.257 W of dynamic power consumption, while the FPGA PL consumes 0.473 W. Within the reconfigurable logic, power is distributed as 0.282 W for the accelerator, 0.099 W for the BRAM, and 0.092 W for the AXI controllers and the reset module.

Table 10 presents the resource utilization of the implemented system on the FPGA. The accelerator uses 36,351 slice registers, 16,100 slice Look-Up Tables (LUTs), 8366 slices, and 65 BRAM tiles. The AXI modules, along with the reset module, utilize 7413 slice registers, 10,278 slice LUTs, and 2993 slices.

Table 11 provides a detailed breakdown of resource usage by module within the accelerator. As shown, slice registers are the most used resources. This high usage is a direct consequence of implementing memory units as register banks to enable parallel reading, as well as from the pipelined comparator trees used for maximum value calculations within each bank. The complete accelerator is distributed over 95.21% of the available slices on the FPGA.

The completely implemented system consumes a total of 1.731 W, with the accelerator itself accounting for only 0.473 W (27% of the total power consumption). The software platform operates on a server-class processor with a TDP of 120–150 W. While TDP represents maximum thermal capacity rather than actual operational power consumption during the sorting task, and the actual CPU power during query strategy execution was not directly measurable in the cloud-based platform, this TDP-based comparison suggests that the proposed system could consume approximately 87× less power under maximum CPU load scenarios. The FPGA accelerator’s 0.473 W consumption represents a potential 317× reduction compared to the processor’s TDP.

The implemented design achieves a processing speed over 200 times faster than the software implementation for the critical case. This speedup results from the in-line selection of the smallest margins using the proposed Algorithm 2, in which the number of clock cycles increases linearly with the number of processed data, in contrast to the quadratic or logarithmic growth seen in traditional sorting-based approaches. This advantage becomes more significant for larger unlabeled datasets, where the cost of instance selection increases, enabling the proposed architecture to exploit its linear scaling behavior fully. However, resource utilization is primarily determined by the batch size, as the architecture necessitates register banks for storage and a comparator tree for each bank. As a result, implementing a batch size at the next power of two ($2^{10}$) is infeasible in the FPGA used in this work. Despite the advantages provided by hardware acceleration, the proposed method has limitations that must be considered. Scalability depends on the volume of data processed, as larger sizes increase the total selection time and pressure on the FPGAs internal resources. Likewise, the use of more complex selection strategies could require additional memory blocks or more comparators, making implementation difficult in devices with limited resources. These limitations point to the need to explore optimizations and more flexible architectures in future work.

To further evaluate our hardware implementation, we benchmarked it against a GPU-based approach. Table 12 reports the metrics of the implementation on an NVIDIA T4 GPU for the margin sampling strategy, including margin calculation and sorting, executed using the CuPy library on Google Colaboratory. This library allows NumPy-type operations to be performed directly on the GPU using CUDA, supported by an efficient memory allocation strategy [73]. To evaluate the system in scenarios of high uncertainty, predictive probabilities were simulated using a Dirichlet distribution (α=200), allowing for the controlled generation of critical classification cases that the algorithm must process [74]. It is possible to observe that margin sampling executes within several hundred milliseconds and consumes approximately 13–18 W of power. The margin sampling and sorting stages use about 52 MiB of VRAM, which corresponds to approximately 0.30% of the 16 GB available on the T4 and about 20 to 26% of the GPU power envelope as reported by nvidia-smi in Google Colaboratory. By comparison, the hardware implementation achieves execution times in the tens of microseconds, resulting in speedups exceeding two orders of magnitude relative to software-based solutions. This substantial reduction in latency, together with the accelerator’s low power consumption, demonstrates the significant computational and energy efficiency advantages of the proposed architecture over GPU-based alternatives. On the other hand, CPU utilization was monitored during execution using the psutil library, and power consumption was estimated proportionally to the observed CPU usage relative to the processor’s maximum power envelope. The reported values correspond to the average execution time, estimated power consumption, and CPU utilization across multiple runs for each dataset.

On the other hand, Table 13 shows the experimental power measurements obtained by monitoring real-time CPU utilization during the selection phase using the Numpy-based baseline. Specifically, power consumption was estimated by recording the average utilization percentage through the psutil library and scaling it against the peak design power (TDP). By moving away from static TDP values, these measurements enable a realistic assessment of the energy-efficiency gains our implementation provides relative to real-world CPU execution. This comparison confirms that our specialized architecture remains significantly more efficient even when the Numpy execution on the CPU operates below its theoretical power limits.

As presented in Table 11, the accelerator exhibits high slice utilization (95.21%) despite moderate LUT (30.40%) and register (34.16%) usage. This discrepancy reveals that the primary bottleneck is routing congestion rather than logic saturation. The distributed register banks and their associated comparator trees create complex interconnection patterns across the FPGA fabric. Each register bank requires connections to its maximum calculation tree, the central comparator, and the output multiplexer, generating extensive routing demands that consume slice resources even when individual logic elements are not fully utilized. This routing-dominated design characteristic limits the feasibility of incorporating additional modules or expanding the batch size on the current device. Integration with other accelerators, such as CNN inference engines, would be challenging given that the margin sampling accelerator already occupies the majority of available routing resources, leaving insufficient flexibility for additional components within the same FPGA.

In the current configuration, where the batch size is set to 10% of the total processed data, scalability is limited, preventing full exploitation of the architecture’s linear complexity advantage. However, design strategies can address these constraints. The most direct approach is to adopt a constant batch size independent of the total dataset size. Under this strategy, the hardware resources remain fixed (e.g., 8 memory units of 64 registers for a batch size of 512). As the unlabeled pool size varies across ALrn iterations, the processing time scales linearly with the current pool size according to (5). For example, processing 4608 samples requires 46.57 μs, while processing 46,080 samples would require approximately 419 μs (at 110 MHz). This approach enables the processing of arbitrarily large datasets within the existing resource constraints while fully leveraging the linear relationship between processing time and dataset size. As the number of samples increases, the processing time grows proportionally (linearly), maintaining a performance advantage over software sorting-based implementations, whose execution time grows logarithmically or quadratically with dataset size.

Regarding the interaction between batch size, approximation quality, and resource constraints, the results of Figure 6 reveal an important scaling property. For a fixed configuration ratio, maintaining the same number of memory units while scaling their size proportionally with batch size, approximation quality improves as batch size grows. For example, the configuration of 8 units of size 128 for a batch size of 1024 produces a more concentrated distribution of mismatched indices near the selection boundary than the configuration of 8 units of size 64 for a batch size of 512 since larger register banks retain more local candidates per unit, reducing the approximation error of the local selection step. Consequently, the approximate nature of the algorithm becomes less pronounced as the problem scales, provided that the pipeline feasibility constraint is satisfied. The primary limitation for larger batch sizes is therefore resource availability on the target device rather than approximation quality degradation. On the current Zynq-7000, batch size 1024 is infeasible due to routing congestion, as the distributed register banks and their associated comparator trees already occupy 95.21% of available slices. Larger devices or Application-Specific Integrated Circuit (ASIC) implementations would relax this constraint, enabling larger batch sizes while maintaining or improving approximation quality.

To further address scalability beyond on-chip memory capacity, the current memory-mapped architecture can be extended to support off-chip data storage through a straightforward integration with AXI DMA. The AXI DMA IP offered by Xilinx provides Memory-Mapped to Stream (MM2S) and Stream to Memory-Mapped (S2MM) interfaces, enabling direct data transfers between off-chip DDR memory and on-chip logic. Since the current architecture exposes a memory-mapped interface, it can be connected to the AXI DMA engine through an AXI4-Stream wrapper, without modifying the core processing logic. Under this scheme, the DMA engine streams chunks of data from DDR into the existing input BRAM, triggers processing, and subsequently retrieves results from the output BRAM back to DDR. This chunked processing approach allows arbitrarily large unlabeled pools to be handled iteratively, with the on-chip BRAM serving as a working buffer rather than requiring full dataset storage. Consequently, the linear scaling property of the accelerator is preserved regardless of the total dataset size.

To validate the efficiency of the proposed hardware, we compared our implementation against previous sorting works developed for the same Xilinx Zynq platform using a pool size of N=4096, which is similar to our critical case [61,62]. As shown in Table 14, our architecture is more than 100× faster than the most efficient state-of-the-art implementation. Notably, while previous works are limited strictly to sorting, our design integrates both the margin sampling calculation and the streaming selection logic. Thus, our implementation handles increasing data volumes much more efficiently, preventing the selection phase from becoming a bottleneck.

A promising direction for improving the system’s scalability is to adopt more flexible architectures. For example, several recent studies have shown that a reconfigurable design on FPGAs enables the accelerator to adapt to different networks and workloads, reducing resource consumption by optimizing the compute and memory structures [26,75]. In addition, memory optimization techniques, such as shared caches or hierarchical schemes, can reduce pressure on on-chip resources, improving efficiency when working with sparse networks or large volumes of data [76]. These strategies illustrate viable paths for implementing future versions of the accelerator with improved scalability and flexibility.

Finally, to provide a system-level perspective, we measured the execution time of a complete ALrn iteration, including training, inference, and query strategy stages. As shown in Table 15, the average execution times indicate that training and inference dominate the overall computation. The query strategy is reported using the best software baseline, corresponding to the proposed algorithm implemented in optimized C++, and compared against the FPGA accelerator. While this stage accounts for only a small fraction of the total time in the evaluated configuration, its computational cost scales with the size of the unlabeled pool, unlike training, which is bounded by the batch size. Therefore, as the unlabeled dataset grows, the cost of instance selection becomes increasingly significant, underscoring the importance of accelerating this stage with specialized hardware.

**Figure 15:**
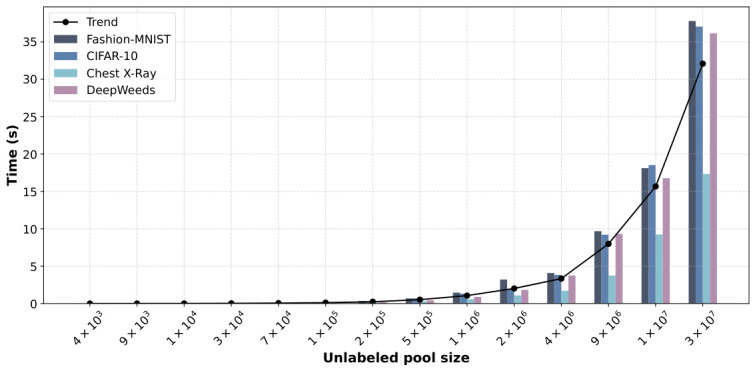
Execution time of the margin sampling query strategy as a function of the unlabeled pool size for different datasets. Results are obtained using a NumPy-based implementation, selecting a fixed batch of 512 samples. As the unlabeled pool size increases, the execution time grows rapidly, highlighting the scalability limitations of software-based implementations. The black line indicates the average trend across datasets.

This trend is further illustrated in Figure 15, where the execution time of margin sampling increases rapidly as the unlabeled pool grows. Therefore, as the unlabeled dataset increases, the cost of instance selection becomes increasingly significant, underscoring the importance of accelerating this stage with specialized hardware.

## 5. Conclusions

In this work, we have presented the design and hardware implementation of a query strategy accelerator for ALrn. The proposed architecture identifies the most uncertain images using the margin between the two highest probabilities for each instance and outputs the corresponding indices for data selection.

The accelerator performs pipelined margin calculation and utilizes multiple memories, as shown in Algorithm 2, for top-*k* selection. This approach preserves the in-line processing nature of the classifier output and achieves a speedup of over two orders of magnitude compared to the software baseline for the critical case. In particular, the accelerator retrieves 512 indices from a pool of 4608 samples in 46.15 μs while the software sorting-based baseline performs the same computation in 10.803 ms. Power measurements in Xilinx Vivado software show a consumption of 1.731 W for the completely implemented system and 0.473 W for the accelerator itself, representing 87× and 317× lower than the 150 W TDP of the software platform, respectively. In comparison, the NVIDIA T4 GPU shows approximately 35× higher power consumption and processing times exceeding two orders of magnitude, further highlighting the energy efficiency and computational advantages of the proposed accelerator.

Scalability is currently constrained by the resource-intensive nature of the architecture. Although individual components (such as FPGA slices) are not fully saturated, the design occupies a large portion of the FPGA fabric, complicating routing and limiting the feasible batch size. In practice, implementing batch sizes at the next power of two would exceed the target device’s available resources. This limitation arises from the fixed-resource mapping inherent in the FPGA design flow, suggesting that ASIC implementations could unlock greater scalability and integration with other accelerators (such as inference engines).

Across all query strategies, the AULC exceeds 65%, reaching approximately 86% in the best cases, while enabling a reduction of 30–40% of the total training data, and even more in some scenarios, to achieve performance levels comparable to those obtained using the full dataset (see Figure 4 and Figure 5). Margin sampling shows a slight advantage (see Table 6), and the hardware-oriented modifications introduced for in-line computation yield negligible deviations with a maximum difference in AULC of 0.0023, suggesting that the proposed selection method can be combined with other query strategies without degrading ALrn performance (see Figure 7 and Table 8). This is particularly relevant when the size of the unlabeled pool grows, as the cost of instance selection and annotation becomes increasingly significant, even when accounting for the full ALrn iteration time (see Table 15). The synergy between ALrn and FPGA-based computation enables the creation of intelligent, efficient, and adaptable systems, especially in embedded and edge applications such as real-time vision [77], anomaly detection [78], low-power autonomous devices [79], precision agriculture [6], and medical applications [5], among others.

The k-selection step is a critical component of the ALrn pipeline, particularly in scenarios involving large unlabeled pools, where uncertainty scores and instance rankings must be computed over thousands or millions of samples. In this context, the proposed architecture replaces conventional sorting-based approaches, typically characterized by logarithmic or quadratic complexity, with a deterministic linear-complexity hardware implementation. This enables efficient and scalable instance selection without degrading ALrn performance, as demonstrated by the negligible deviation in the learning curves. Furthermore, the proposed approach is not limited to ALrn but can be extended to any application requiring efficient top-*k* selection under resource constraints.

Future work will explore hybrid strategies that combine uncertainty criteria (e.g., margin sampling) with diversity metrics to select more representative data, where the FPGA can calculate uncertainty scores in parallel with low-cost diversity metrics, enabling joint selection without significantly increasing latency. Likewise, heterogeneous memory approaches will be considered by allocating memory blocks of different sizes within the architecture, allowing intermediate values, partial scores, or candidate indices to be stored in units of appropriate size. Future work will also examine how clock frequency scaling affects performance and power consumption to more accurately characterize the trade-offs between efficiency and performance of the proposed accelerator. These guidelines represent feasible implementation paths consistent with current design practices in FPGA. In addition, evaluating the accelerator in real-world scenarios and with higher-resolution images will allow for analyzing behavior under more demanding conditions and expand its applicability in practical implementations. Finally, complementary evaluations on modern low-power edge–AI platforms such as NVIDIA Jetson, Google Coral, or microcontroller-oriented ML devices will provide a broader perspective on how the proposed FPGA accelerator compares with other embedded inference solutions.

## Figures and Tables

**Table 1:** Comparison of hardware approaches related to classification, sorting mechanisms, and Active Learning.

Work	Classification Tasks	Sorting/Top-*k*	Active Learning	AL Query Strategy
Kim et al. (2025) [29]	√	×	×	×
Xu et al. (2024) [26]	√	×	×	×
Ben Jmaa et al. (2019, 2024) [61,62]	×	√	×	×
Aydin and Bilge (2023) [25]	√	×	×	×
Ebrahim and Khalifat (2023) [28]	×	√	×	×
Ours	√	√	√	√

**Table 2:** Summary of datasets used in this study, including resolution, images per class, and categories.

Dataset	Resolution (px)	Images/Class	Categories
Fashion-MNIST	28 × 28	7000	T-shirt/top, Trouser, Pullover, Dress, Coat, Sandal, Shirt, Sneaker, Bag, Ankle Boot
CIFAR-10	32 × 32	6000	Airplane, Automobile, Bird, Cat, Deer, Dog, Frog, Horse, Ship, Truck
Chest X-Ray	**—**	Imbalanced	COVID-19 (576), Normal (1583), Pneumonia (4273)
DeepWeeds	256 × 256	Imbalanced	Chinee apple (1125), Lantana (1064), Parkinsonia (1031), Parthenium (1022), Prickly acacia (1062), Rubber vine (1009), Siam weed (1074), Snake weed (1016), Negatives (9106)

**—** indicates that the dataset contains images with varying resolutions, ranging from 127 × 382 to 4757 × 5623 pixels.

**Table 3:** Total and trainable parameter counts for each CNN architecture employed.

Architecture	Total Params	Trainable Params
MobileNetV1	3,239,114	10,250
EfficientNetB0	4,062,381	12,810
ResNet50	23,608,202	**20,490**

**Bolded** value represents the highest quantity of trainable parameters.

**Table 4:** Training hyperparameters used in all classifiers for all experiments.

Hyperparameter	Value
Learning rate	0.001
Batch size	32
Max epoch	100
Optimizer	Adam
Error function	Cross-entropy

**Table 5:** Classification performance of different CNNs on CIFAR-10, Fashion-MNIST, Chest X-Ray and DeepWeeds datasets, evaluated using ACC and F1.

CNN	CIFAR-10	Fashion-MNIST	Chest X-Ray	DeepWeeds
**ACC**				
MobileNetV1	84.17	87.35	95.82	87.35
EfficientNetB0	**88.25**	**89.28**	95.46	**89.28**
ResNet50	87.23	87.40	**96.20**	87.40
**F1**				
MobileNetV1	84.16	87.23	95.85	87.23
EfficientNetB0	**88.25**	**89.18**	95.51	**89.18**
ResNet50	87.28	87.42	**96.21**	87.42

**Bolded** values indicate the highest performance achieved for each dataset and evaluation metric.

**Table 6:** AULC results for ACC and F1 across query strategies and classifiers on the Fashion-MNIST, CIFAR-10, Chest X-Ray and DeepWeeds datasets.

CNN	ACC				F1			
	RND	LC	MRG	ENT	RND	LC	MRG	ENT
**Fashion-MNIST**								
MobileNetV1	0.7718	0.7776	**0.7785**	0.7770	0.7706	0.7765	**0.7774**	0.7759
EfficientNetB0	0.7881	0.7956	**0.7965**	0.7931	0.7870	0.7946	**0.7954**	0.7921
ResNet50	0.7688	0.7781	**0.7781**	0.7778	0.7679	0.7764	**0.7765**	0.7762
**CIFAR-10**								
MobileNetV1	0.7393	0.7486	**0.7493**	0.7474	0.7387	0.7484	**0.7492**	0.7474
EfficientNetB0	0.7800	0.7882	**0.7884**	0.7876	0.7796	0.7880	**0.7883**	0.7875
ResNet50	0.7734	0.7813	**0.7815**	0.7803	0.7730	0.7811	**0.7813**	0.7802
**Chest X-Ray**								
MobileNetV1	0.8582	0.8614	0.8619	**0.8622**	0.8582	0.8614	0.8618	**0.8622**
EfficientNetB0	0.8569	0.8630	**0.8638**	0.8623	0.8569	0.8630	**0.8637**	0.8623
ResNet50	0.8546	**0.8635**	0.8627	0.8628	0.8543	**0.8635**	0.8627	0.8627
**DeepWeeds**								
MobileNetV1	0.6725	0.6785	**0.6826**	0.6782	0.6712	0.6769	**0.6810**	0.6771
EfficientNetB0	0.7264	0.7395	**0.7394**	0.7388	0.7239	0.7373	**0.7374**	0.7369
ResNet50	0.7118	**0.7262**	0.7256	0.7207	0.7102	**0.7248**	0.7239	0.7187

**Bolded** values indicate the strategy with the highest AULC in each row for a given performance metric. Query strategies: RND = Random, LC = Least Confident, MRG = Margin, ENT = Entropy.

**Table 7:** Mismatched index counts across memory configurations. Each value represents the mean (over 100 permutations) of the minimum, maximum, average, and standard deviation of the number of indices selected by the proposed algorithm but not in the baseline top-10%.

Unit	Size	Statistics			
		Min	Max	Avg	Std
**Number of data: 2560**					
2	128	55.00	76.00	65.30	4.78
4	64	37.00	58.00	46.75	5.01
8	32	24.00	45.00	32.83	4.90
16	16	14.00	39.00	23.85	5.29
32	8	4.00	28.00	15.91	4.78
**Number of data: 5120**					
2	256	113.00	149.00	130.96	6.69
4	128	80.00	119.00	93.77	7.22
8	64	55.00	88.00	67.12	6.01
16	32	31.00	64.00	47.63	6.41
32	16	18.00	51.00	33.39	6.46
64	8	10.00	44.00	22.29	6.83
**Number of data: 10,240**					
2	512	237.00	283.00	260.71	9.18
4	256	164.00	206.00	188.40	9.36
8	128	109.00	164.00	134.18	9.49
16	64	67.00	117.00	93.76	8.66
32	32	47.00	89.00	67.64	8.09
64	16	26.00	72.00	45.89	8.94
128	8	10.00	65.00	32.02	8.84

**Unit**: number of memory units; **Size:** size of each memory unit. Min, Max, Avg, and Std represent the mean minimum, mean maximum, mean average, and mean standard deviation of mismatched index counts across 100 permutations for each memory configuration.

**Table 8:** AULC results for ACC and F1 using margin sampling, comparing the baseline and the emulated multiple memories approach across classifiers on the Fashion-MNIST, CIFAR-10, DeepWeeds, and Chest X-Ray datasets.

Configuration	ACC		F1	
	Baseline	Emulation	Baseline	Emulation
MobileNetV1 on Fashion-MNIST	0.7785	0.7792	0.7774	0.7783
MobileNetV1 on CIFAR-10	0.7493	0.7495	0.7492	0.7495
MobileNetV1 on Chest X-Ray	0.8619	0.8613	0.8618	0.8614
MobileNetV1 on DeepWeeds	**0.6826**	**0.6803**	**0.6810**	**0.6791**
EfficientNetB0 on Fashion-MNIST	0.7965	0.7967	0.7954	0.7956
EfficientNetB0 on CIFAR-10	**0.7884**	**0.7885**	**0.7883**	**0.7884**
EfficientNetB0 on Chest X-Ray	0.8638	0.8633	0.8637	0.8633
EfficientNetB0 on DeepWeeds	0.7394	0.7406	0.7374	0.7387
ResNet50 on Fashion-MNIST	0.7781	**0.7765**	0.7765	0.7752
ResNet50 on CIFAR-10	0.7815	0.7812	0.7813	0.7810
ResNet50 on Chest X-Ray	0.8627	0.8631	0.8627	0.8630
ResNet50 on DeepWeeds	0.7256	0.7247	0.7239	0.7231

**Bolded** values highlight notable differences between baseline and emulated approaches. The largest difference occurs for MobileNetV1 on DeepWeeds (ACC difference of 0.0023), while the smallest difference is observed for EfficientNetB0 on CIFAR-10 (ACC and F1 differences of 0.0001).

**Table 9:** Execution time comparison across software implementations and hardware accelerator for the critical case ($N_{data}=4608$, batch size =512). Software times represent the mean of 1000  independent runs. The PL-only hardware time is estimated via (5) at 110 MHz. The end-to-end hardware time includes PS→PL transfer, PL processing, and PL→PS transfer, measured over 50 independent runs using direct memory-mapped I/O from a C program on the ARM processor.

Platform	Algorithm	Time (ms)	vs. Baseline	vs. Best SW
Python	Sorting-based	10.803	1× (baseline)	—
Python	Proposed	44.406	0.24×	—
C++	Sorting-based	1.646	6.6×	—
C++	Proposed	1.068	10.1×	1× (best)
Hardware (FPGA)	Proposed (PL only)	0.04661	231.7×	22.9×
Hardware (FPGA)	Proposed (end-to-end)	15.098	0.72×	0.07×

**Table 10:** FPGA resource utilization summary for the complete implemented system, including the accelerator and AXI modules with reset controller. The reported values correspond to post-implementation resource usage as estimated by Xilinx Vivado 2024.2.

	Slice LUTs	Slice Registers	Slices	Block RAM Tiles
AXIs/Reset	10,278	7413	2993	0
Accelerator	16,160	36,351	8366	65
Total	23,565	46,629	11,239	65
Available	53,200	106,400	13,300	140
Percentage	44.38%	43.82%	88.17%	46.43%

**Table 11:** Detailed resource utilization breakdown for each module within the hardware accelerator. Slice registers represent the most heavily used resource due to the implementation of memory units as register banks for parallel reading and the use of pipelined maximum comparator trees.

	Slice LUTs	Slice Registers	Slices	Block RAM Tiles
Controller	48	13	36	0
Counters	43	100	53	0
Multiplexers	1781	0	476	0
Margin tree	360	467	113	0
Registers	620	208	501	0
Register banks	4609	17,920	7390	0
Max trees	8496	17,640	3959	0
Comparator	0	0	2	0
BRAMs	220	2	133	65
Total	16,177	36,350	12,663	65
Available	53,200	106,400	13,300	140
Percentage	30.40%	34.16%	95.21%	46.43%

**Table 12:** Time, power, and memory utilization for the margin sampling stage executed on an NVIDIA T4 GPU and the proposed FPGA-based accelerator.

Platform/Dataset	Time [s]	Power [W]	Memory Utilization
NVIDIA T4—DeepWeeds	0.297135	16.985	52 MiB (0.32% of VRAM)
NVIDIA T4—ChestX-Ray	0.279612	13.875	52 MiB (0.32% of VRAM)
NVIDIA T4—CIFAR-10	0.279149	17.725	52 MiB (0.32% of VRAM)
NVIDIA T4—Fashion-MNIST	0.350990	18.460	52 MiB (0.32% of VRAM)
NVIDIA T4—Mean	0.301722	16.761	52 MiB (0.32% of VRAM)
Proposed FPGA accelerator *	$46.15\times 10^{-6}$	0.473	65 BRAM tiles (46.43% of BRAM)

* The most critical case, i.e., a digital system with ten classes.

**Table 13:** Time, power, and CPU utilization for the margin sampling stage executed on an Intel Xeon CPU (Numpy baseline) and our implementation.

Platform/Dataset	Time [s]	Power [W]	CPU Utilization
Intel Xeon—DeepWeeds	0.000398	30.00	25.00%
Intel Xeon—ChestX-Ray	0.000326	30.00	25.00%
Intel Xeon—CIFAR-10	0.000640	19.98	16.65%
Intel Xeon—Fashion-MNIST	0.000397	60.00	50.00%
Intel Xeon—Mean	0.000440	34.99	29.16%
Our implementation *	$46.15\times 10^{-6}$	0.473	65 BRAM tiles (46.43%)

* The most critical case, i.e., a digital system with ten classes.

**Table 14:** Execution time comparison against state-of-the-art sorting implementations.

Algorithm	Execution Time [μs]
BubbleSort	336,356.0
SelectionSort	168,618.0
InsertionSort	168,554.0
QuickSort	38,599.0
MergeSort	4812.4
Proposed Accelerator	46.15

**Table 15:** Average execution time of a complete Active Learning iteration. Query strategy times are reported for software (optimized C++) and hardware (FPGA, PL processing only).

Model	Dataset	Training (s)	Inference (s)	Implementations	
				**Software (s)**	**Hardware (s)**
ResNet50	Fashion-MNIST	81.60	27.70	–	–
	CIFAR-10	82.29	28.17	–	–
	Chest X-Ray	98.97	27.16	–	–
	DeepWeeds	86.53	29.42	–	–
MobileNet	Fashion-MNIST	45.06	13.30	–	–
	CIFAR-10	48.24	13.28	–	–
	Chest X-Ray	76.05	14.47	–	–
	DeepWeeds	47.38	14.05	–	–
EfficientNet	Fashion-MNIST	73.51	14.09	–	–
	CIFAR-10	76.60	13.36	–	–
	Chest X-Ray	83.53	13.59	–	–
	DeepWeeds	90.21	13.14	–	–
Mean		74.16	18.48	0.001068	0.0000466

## Data Availability

All datasets used in this study are publicly available (accessed on 31 March 2026). Fashion-MNIST is provided at https://github.com/zalandoresearch/fashion-mnist, CIFAR-10 at https://www.cs.toronto.edu/~kriz/cifar.html, the Chest X-Ray dataset at https://www.kaggle.com/datasets/prashant268/chest-xray-covid19-pneumonia, and the DeepWeeds dataset at https://github.com/AlexOlsen/DeepWeeds. All code developed for this study, including hardware description files and notebooks for data processing and model training, is publicly accessible in the project repository at https://github.com/abarbierif/al_hw. Researchers are encouraged to use these resources to reproduce and extend the results reported in this work.

## Abbreviations

The following abbreviations are used in this manuscript:

**Parameter**	**Definition**
ACC	Accuracy
AI	Artificial Intelligence
ALrn	Active Learning
ALU	Arithmetic Logic Unit
ARM	Advanced RISC Machine
ASIC	Application-Specific Integrated Circuit
AULC	Area Under the Learning Curve
AXI	Advanced eXtensible Interface
BGR	Blue, Green, and Red
BRAM	Block Random Access Memory
CNN	Convolutional Neural Network
CPU	Central Processing Unit
DDR	Double Data Rate
DL	Deep Learning
DNN	Deep Neural Network
F1	F1-score
FPGA	Field Programmable Gate Array
GPIO	General-Purpose Input/Output
GPU	Graphics Processing Unit
LUT	Look-Up Table
ML	Machine Learning
PLrn	Passive Learning
PL	Programmable Logic
PS	Processing System
PYNQ	Python Productivity for Zynq
RGB	Red, Green and Blue
SoC	System on Chip
TDP	Thermal Design Power
ViT	Vision Transformers

## Appendix A

This appendix provides a detailed comparison of ALrn algorithms across different CNN models and datasets. Table A1, Table A2, Table A3, Table A4, Table A5, Table A6, Table A7, Table A8, Table A9, Table A10, Table A11 and Table A12 report the minimum number of labeled training examples required for each query strategy to achieve a specific performance in terms of ACC and F1. Results are presented separately for Fashion-MNIST, CIFAR-10, Chest X-Ray, and DeepWeeds as well as for each of the three evaluated CNN architectures: MobileNetV1, EfficientNetB0, and ResNet50. This analysis complements the learning curves discussed in Section 3.3 of the main text.

**Table A1:** Minimum number of training examples required by each Active Learning algorithm to reach the target ACC and F1 metrics on the Fashion-MNIST dataset using the MobileNetV1 CNN.

ACC (%)	RND	LC	MRG	ENT	F1 (%)	RND	LC	MRG	ENT
76.42	—	—	—	—	73.93	—	—	—	—
76.52	512	512	512	512	74.03	512	512	512	512
76.62	1024	1024	1024	1024	74.13	1024	1024	1024	1024
83.42	1024	1024	1024	1536	83.43	1024	1024	1024	1536
84.12	1536	1024	1024	1536	84.13	1536	1024	1024	1536
84.42	1536	1536	**1024**	1536	84.53	1536	1536	**1024**	1536
84.52	1536	1536	1536	1536	84.73	2048	1536	1536	1536
84.72	2048	1536	1536	1536	85.73	2048	1536	1536	2048
85.62	2048	1536	1536	2048	85.83	2560	1536	1536	2048
85.82	2560	1536	1536	2048	86.13	2560	**1536**	2048	2048
86.02	2560	**1536**	2048	2048	86.23	3072	**1536**	2048	2048
86.22	3072	**1536**	2048	2048	86.63	3072	2048	2048	2048
86.62	3072	2560	2048	2048	86.73	3072	2560	2048	2048
86.82	3072	2560	2560	**2048**	86.93	3072	2560	2560	2560
86.92	3072	2560	2560	2560	87.13	4096	2560	2560	2560
87.12	3584	2560	2560	2560	87.43	4608	3584	2560	2560
87.22	4096	2560	2560	2560	87.63	—	3584	3584	**3072**
87.42	4096	3584	2560	2560	87.73	—	3584	3584	3584
87.52	4608	3584	2560	2560	87.93	—	4608	3584	3584
87.62	—	3584	3584	**3072**	88.03	—	—	3584	3584
87.72	—	3584	3584	3584	88.23	—	—	4096	**3584**
88.02	—	—	3584	3584	88.33	—	—	—	**3584**
88.22	—	—	4096	**3584**	88.47	—	—	—	—
88.32	—	—	—	**3584**					
88.44	—	—	—	—					

**Bolded** values indicate the minimum number of data samples required when smaller than in the other strategies. The symbol “—” denotes that the specified performance in the row was not achieved. Query strategies: RND = Random, LC = Least Confident, MRG = Margin, ENT = Entropy.

**Table A2:** Minimum number of training examples required by each Active Learning algorithm to reach the target ACC and F1 metrics on the Fashion-MNIST dataset using the EfficientNetB0 CNN.

ACC (%)	RND	LC	MRG	ENT	F1 (%)	RND	LC	MRG	ENT
78.46	—	—	—	—	76.33	—	—	—	—
78.56	512	512	512	512	76.43	512	512	512	512
78.66	1024	**512**	1024	1024	76.53	1024	**512**	1024	1024
78.96	1024	1024	1024	1024	76.93	1024	1024	1024	1024
85.16	1024	1024	1024	1536	85.23	1024	1024	1024	1536
85.46	1536	1024	1024	1536	85.53	1536	1024	1024	1536
86.06	1536	1536	**1024**	1536	86.13	1536	1536	**1024**	1536
86.46	1536	1536	1536	1536	86.53	1536	1536	1536	1536
86.96	2048	1536	1536	1536	87.03	2048	1536	1536	1536
87.56	2560	1536	1536	1536	87.63	2560	1536	1536	1536
87.66	2560	1536	1536	2048	87.73	2560	1536	1536	2048
88.06	3072	1536	1536	2048	88.03	3072	1536	1536	2048
88.36	3072	2048	**1536**	2048	88.43	3072	2048	**1536**	2048
88.56	3584	2048	2048	2048	88.53	3584	2048	**1536**	2048
88.86	3584	2048	2048	2560	88.63	3584	2048	2048	2048
89.16	4608	2560	**2048**	2560	88.83	3584	2048	2048	2560
89.46	4608	3072	2560	2560	89.13	4608	2048	2048	2560
89.66	—	3072	3072	4096	89.23	4608	2560	**2048**	2560
89.76	—	3072	3072	—	89.43	4608	3072	2560	2560
89.86	—	—	**3072**	—	89.63	—	3072	3072	4096
90.03	—	—	—	—	89.83	—	—	**3072**	—
					89.94	—	—	—	—

**Bolded** values indicate the minimum number of data samples required when smaller than in the other strategies. The symbol “—” denotes that the specified performance in the row was not achieved. Query strategies: RND = Random, LC = Least Confident, MRG = Margin, ENT = Entropy.

**Table A3:** Minimum number of training examples required by each Active Learning algorithm to reach the target CNN and F1 metrics on the Fashion-MNIST dataset using the ResNet50 CNN.

ACC (%)	RND	LC	MRG	ENT	F1 (%)	RND	LC	MRG	ENT
76.59	—	—	—	—	73.87	—	—	—	—
76.69	512	512	512	512	73.97	512	512	512	512
76.79	1024	1024	1024	1024	74.07	1024	1024	1024	1024
83.49	1536	1024	1024	1536	83.47	1024	1024	1024	1536
83.69	1536	1536	**1024**	1536	83.57	1536	1024	1024	1536
84.39	1536	1536	1536	1536	83.67	1536	1536	**1024**	1536
84.79	2560	1536	1536	1536	84.47	1536	1536	1536	1536
85.89	2560	2048	1536	1536	84.87	2048	1536	1536	1536
85.99	3072	2048	1536	1536	85.07	2560	1536	1536	1536
86.29	3072	2048	2048	**1536**	85.97	2560	2048	1536	1536
86.59	3584	2048	2048	**1536**	86.07	3072	2048	1536	1536
86.69	3584	2048	2048	2048	86.37	3072	2048	2048	**1536**
86.79	4096	2048	2048	2048	86.47	3584	2048	2048	**1536**
86.89	4096	2560	2048	2048	86.87	3584	2048	2048	2048
87.09	4608	2560	2560	**2048**	86.97	4096	2560	2048	2048
87.19	4608	2560	2560	2560	87.07	4608	2560	2560	**2048**
87.29	4608	2560	3072	2560	87.27	4608	2560	2560	2560
87.49	4608	3072	3072	3072	87.37	4608	2560	3072	2560
87.59	—	**3072**	3584	4096	87.47	4608	**3072**	3584	3584
87.79	—	**3072**	3584	4608	87.57	—	**3072**	3584	4096
87.89	—	3584	3584	4608	87.77	—	3584	3584	4608
87.99	—	3584	3584	5120	87.97	—	3584	3584	—
88.09	—	**3584**	—	—	88.07	—	**4608**	—	—
88.19	—	**4608**	—	—	88.26	—	—	—	—
88.28	—	—	—	—					

**Bolded** values indicate the minimum number of data samples required when smaller than in the other strategies. The symbol “—” denotes that the specified performance in the row was not achieved. Query strategies: RND = Random, LC = Least Confident, MRG = Margin, ENT = Entropy.

**Table A4:** Minimum number of training examples required by each Active Learning algorithm to reach the target ACC and F1 metrics on the CIFAR-10 dataset using the MobileNetV1 CNN.

ACC (%)	RND	LC	MRG	ENT	F1 (%)	RND	LC	MRG	ENT
75.85	—	—	—	—	75.75	—	—	—	—
75.95	512	512	512	512	75.85	512	512	512	512
76.05	1024	1024	1024	1024	75.95	1024	1024	1024	1024
79.75	1536	1024	1024	1024	79.65	1536	1024	1024	1024
80.55	1536	1536	**1024**	1536	80.55	1536	1536	1024	1024
81.05	2048	1536	**1024**	1536	80.65	1536	1536	**1024**	1536
81.15	2048	1536	1536	1536	80.85	2048	1536	**1024**	1536
81.85	2560	1536	1536	1536	81.15	2048	1536	1536	1536
81.95	2560	1536	1536	2048	81.85	2560	1536	1536	1536
82.35	3072	**1536**	2048	2048	81.95	2560	1536	1536	2048
82.45	3072	2048	2048	2048	82.25	3072	1536	1536	2048
83.05	3072	2560	2048	2048	82.35	3072	**1536**	2048	2048
83.15	3072	2560	2560	**2048**	82.45	3072	2048	2048	2048
83.45	3584	2560	2560	**2048**	83.05	3072	2560	2048	2048
83.55	3584	2560	3072	**2048**	83.15	3072	2560	2560	**2048**
83.75	4096	2560	3072	**2048**	83.35	3584	2560	2560	**2048**
83.85	5120	**2560**	3072	3072	83.55	3584	2560	3072	**2048**
83.95	5120	3072	3072	3072	83.65	4096	2560	3072	**2048**
84.05	5120	3072	3072	3584	83.85	5120	3072	3072	**2048**
84.25	—	3072	3072	3584	83.95	5120	3072	3072	3584
84.45	—	**3072**	3584	3584	84.35	—	**3072**	**3072**	3584
84.55	—	4096	3584	3584	84.45	—	3584	3584	3584
84.65	—	4608	3584	3584	84.55	—	4096	3584	3584
84.85	—	5120	4096	**3584**	84.65	—	4608	3584	3584
84.95	—	5120	5120	—	84.85	—	5120	4096	**3584**
85.05	—	—	5120	—	84.95	—	5120	5120	—
85.42	—	—	**5120**	—	85.05	—	—	5120	—
					85.46	—	—	**5120**	—

**Bolded** values indicate the minimum number of data samples required when smaller than in the other strategies. The symbol “—” denotes that the specified performance in the row was not achieved. Query strategies: RND = Random, LC = Least Confident, MRG = Margin, ENT = Entropy.

**Table A5:** Minimum number of training examples required by each Active Learning algorithm to reach the target ACC and F1 metrics on the CIFAR-10 dataset using the EfficientNetB0 CNN.

ACC (%)	RND	LC	MRG	ENT	F1 (%)	RND	LC	MRG	ENT
81.95	—	—	—	—	81.89	—	—	—	—
82.05	512	512	512	512	81.99	512	512	512	512
82.15	1024	512	512	512	82.09	1024	512	512	512
82.25	1024	1024	1024	1024	82.19	1024	1024	1024	1024
84.45	1536	1024	1024	1024	84.39	1536	1024	1024	1024
85.05	1536	**1024**	**1024**	1536	85.09	1536	**1024**	**1024**	1536
85.35	1536	1536	1536	1536	85.29	1536	1536	1536	1536
85.65	2048	1536	1536	1536	85.59	2048	1536	1536	1536
86.25	2560	1536	1536	1536	86.19	2560	1536	1536	1536
86.65	2560	1536	1536	2048	86.69	2560	**1536**	2048	2048
86.75	2560	**1536**	2048	2048	86.89	2560	2048	2048	2048
86.85	2560	2048	2048	2048	87.39	3072	2048	2048	2048
87.35	3072	2048	2048	2048	87.49	3584	2048	2048	2048
87.55	3584	2048	2048	2048	87.59	4096	2048	2048	2560
87.65	4096	**2048**	**2048**	2560	87.79	4096	2560	**2048**	2560
87.85	4096	2560	2560	2560	87.89	4096	2560	2560	2560
88.05	4608	2560	2560	3072	88.09	4608	2560	2560	3072
88.15	4608	3072	**2560**	3072	88.19	5120	3072	**2560**	3072
88.25	5120	3072	**2560**	3072	88.39	5120	3072	3072	3072
88.45	5120	3072	3072	3072	88.59	—	3584	**3072**	**3072**
88.65	—	4096	**3584**	**3584**	88.69	—	4096	**3584**	**3584**
88.75	—	4096	4096	4096	88.79	—	4096	4096	4096
88.85	—	4096	—	4096	88.89	—	**4096**	—	—
88.95	—	**4096**	—	—	89.06	—	—	—	—
89.06	—	—	—	—					

**Bolded** values indicate the minimum number of data samples required when smaller than in the other strategies. The symbol “—” denotes that the specified performance in the row was not achieved. Query strategies: RND = Random, LC = Least Confident, MRG = Margin, ENT = Entropy.

**Table A6:** Minimum number of training examples required by each Active Learning algorithm to reach the target ACC and F1 metrics on the CIFAR-10 dataset using the ResNet50 CNN.

ACC (%)	RND	LC	MRG	ENT	F1 (%)	RND	LC	MRG	ENT
80.09	—	—	—	—	79.96	—	—	—	—
80.19	512	512	512	512	80.06	512	512	512	512
80.29	1024	1024	1024	1024	80.16	1024	1024	1024	1024
83.79	1536	1024	1024	1024	83.66	1536	1024	1024	1024
84.49	1536	1024	1024	1536	84.56	1536	1024	1024	1536
84.79	1536	1536	**1024**	1536	84.76	1536	1536	**1024**	1536
84.89	1536	1536	1536	1536	84.86	1536	1536	1536	1536
85.09	1536	1536	2048	1536	84.96	1536	1536	2048	1536
85.19	2048	1536	2048	1536	85.06	2048	1536	2048	1536
85.89	2560	**1536**	2048	2048	85.86	2560	**1536**	2048	2048
85.99	2560	2048	2048	2048	85.96	2560	2048	2048	2048
86.09	3072	2048	2048	2048	86.06	3072	2048	2048	2048
86.59	3072	2048	2048	2560	86.46	3072	2048	2048	2560
86.79	3584	2048	2048	2560	86.76	3584	2048	2048	2560
87.09	3584	2560	**2048**	2560	86.96	3584	2560	**2048**	2560
87.29	4096	2560	**2048**	3072	87.26	4096	2560	**2048**	2560
87.39	4096	2560	2560	3072	87.36	4096	2560	2560	3072
87.49	4608	3072	**2560**	3072	87.46	4608	3072	**2560**	3072
87.59	—	3072	**2560**	3072	87.56	—	3072	**2560**	3072
87.79	—	3072	3584	3072	87.86	—	3072	3584	3072
88.19	—	5120	3584	**3072**	88.16	—	**3072**	3584	—
88.29	—	5120	**4096**	—	88.26	—	5120	**4096**	—
88.39	—	5120	—	—	88.46	—	5120	—	—
88.48	—	**5120**	—	—	88.47	—	**5120**	—	—

**Bolded** values indicate the minimum number of data samples required when smaller than in the other strategies. The symbol “—” denotes that the specified performance in the row was not achieved. Query strategies: RND = Random, LC = Least Confident, MRG = Margin, ENT = Entropy.

**Table A7:** Minimum number of training examples required by each Active Learning algorithm to reach the target ACC and F1 metrics on the Chest X-Ray dataset using the MobileNetV1 CNN.

ACC (%)	RND	LC	MRG	ENT	F1 (%)	RND	LC	MRG	ENT
93.81	—	—	—	—	93.76	—	—	—	—
93.91	512	512	512	512	93.86	512	512	512	512
94.01	1024	1024	512	512	93.96	1024	1024	512	512
94.11	1024	1024	**512**	1024	94.06	1024	1024	**512**	1024
94.21	1024	1024	1024	1024	94.16	1024	1024	1024	1024
94.91	1536	1024	1024	1024	94.96	1536	1024	1024	1024
95.31	2560	1024	1024	1536	95.26	2560	1024	1024	1536
95.51	3072	**1024**	1536	1536	95.56	3072	1536	1536	1536
95.61	3072	1536	1536	1536	95.66	3072	1536	2048	1536
95.71	4096	2048	2048	**1536**	95.76	4096	2048	2048	**1536**
95.91	—	2048	2048	**1536**	95.86	—	2048	2048	**1536**
96.11	—	—	**2048**	3072	96.06	—	2048	2048	2048
96.21	—	—	**2048**	—	96.16	—	—	**2048**	—
96.23	—	—	—	—	96.22	—	—	—	—

**Bolded** values indicate the minimum number of data samples required when smaller than in the other strategies. The symbol “—” denotes that the specified performance in the row was not achieved. Query strategies: RND = Random, LC = Least Confident, MRG = Margin, ENT = Entropy.

**Table A8:** Minimum number of training examples required by each Active Learning algorithm to reach the target ACC and F1 metrics on the Chest X-Ray dataset using the EfficientNetB0 CNN.

ACC (%)	RND	LC	MRG	ENT	F1 (%)	RND	LC	MRG	ENT
93.01	—	—	—	—	93.0	—	—	—	—
93.11	512	512	512	512	93.1	512	512	512	512
93.21	512	512	512	1024	93.2	512	512	512	1024
93.31	1024	512	512	1024	93.3	1024	512	512	1024
93.51	1024	1024	**512**	1024	93.5	1024	1024	1024	1024
93.61	1024	1024	1024	1024	94.1	1536	1024	1024	1024
94.01	1536	1024	1024	1024	94.9	2048	1024	1024	1024
94.91	2048	1024	1024	1024	95.0	2048	1024	1024	1536
95.01	2048	1024	1024	1536	95.4	3584	1536	**1024**	1536
95.41	3584	1536	**1024**	1536	95.5	3584	1536	1536	1536
95.51	3584	1536	1536	1536	95.6	4096	1536	1536	1536
95.61	4096	1536	1536	1536	95.9	4608	1536	1536	1536
95.91	4608	1536	1536	1536	96.0	4608	1536	1536	2048
96.01	4608	1536	1536	2048	96.1	4608	**1536**	2048	2048
96.11	5120	**1536**	2048	2048	96.2	5120	2048	2048	2048
96.21	5120	2048	2048	2048	96.3	—	**2048**	—	2560
96.31	—	3584	—	**2560**	96.4	—	—	—	**2560**
96.41	—	—	—	—	96.4	—	—	—	—

**Bolded** values indicate the minimum number of data samples required when smaller than in the other strategies. The symbol “—” denotes that the specified performance in the row was not achieved. Query strategies: RND = Random, LC = Least Confident, MRG = Margin, ENT = Entropy.

**Table A9:** Minimum number of training examples required by each Active Learning algorithm to reach the target ACC and F1 metrics on the Chest X-Ray dataset using the ResNeto50 CNN.

ACC (%)	RND	LC	MRG	ENT	F1 (%)	RND	LC	MRG	ENT
93.01	—	—	—	—	92.98	—	—	—	—
93.11	512	512	512	512	93.08	512	512	512	512
93.21	512	512	1024	512	93.18	512	512	1024	512
93.51	1024	512	1024	512	93.48	1024	512	1024	512
93.61	1024	512	1024	1024	93.68	1024	512	1024	1024
93.81	1024	1024	1024	1024	93.78	1024	1024	1024	1024
94.11	1536	1024	1024	1024	93.98	1536	1024	1024	1024
94.61	2560	1024	1024	1024	94.48	2560	1024	1024	1024
95.41	3584	1024	1024	1024	95.38	3584	1024	1024	1024
95.51	4096	1024	1024	1024	95.48	4096	1024	1024	1024
95.61	4096	1024	1536	1024	95.68	4096	1024	1536	1024
95.71	4096	1536	1536	**1024**	95.78	—	1536	1536	1536
95.81	—	1536	1536	1536	96.28	—	1536	4096	1536
96.21	—	1536	2048	1536	96.38	—	1536	—	—
96.31	—	1536	4096	1536	96.44	—	—	—	—
96.41	—	1536	—	—					
96.45	—	—	—	—					

**Bolded** values indicate the minimum number of data samples required when smaller than in the other strategies. The symbol “—” denotes that the specified performance in the row was not achieved. Query strategies: RND = Random, LC = Least Confident, MRG = Margin, ENT = Entropy.

**Table A10:** Minimum number of training examples required by each Active Learning algorithm to reach the target ACC and F1 metrics on the DeepWeeds dataset using the MobileNetV1 CNN.

ACC (%)	RND	LC	MRG	ENT	F1 (%)	RND	LC	MRG	ENT
65.24	—	—	—	—	64.96	—	—	—	—
65.34	512	512	512	512	65.06	512	512	512	512
65.44	512	1024	512	512	65.16	512	1024	512	512
65.74	512	1024	1024	1024	65.56	512	1024	1024	1024
66.34	1024	1024	1024	1024	65.96	1024	1024	1024	1024
70.94	1024	1024	1024	1536	70.76	1536	1024	1024	1024
71.04	1536	1024	1024	1536	70.96	1536	1536	1024	1536
71.14	1536	1536	**1024**	1536	71.86	1536	1536	1536	1536
71.94	1536	1536	1536	1536	72.56	2048	1536	1536	1536
72.74	2048	1536	1536	1536	73.16	2048	2048	**1536**	1536
73.44	2048	2048	**1536**	2048	73.46	2048	2048	**1536**	2048
74.14	2560	2048	**1536**	2048	74.06	2560	2048	**1536**	2048
74.44	2560	2048	2048	2048	74.36	2560	2048	2048	2048
74.84	2560	2560	2048	2048	74.56	2560	2560	2048	2048
74.94	2560	2560	**2048**	2560	74.96	2560	2560	**2048**	2560
75.84	3072	2560	**2048**	2560	75.66	3072	2560	**2048**	2560
75.94	3072	2560	2560	2560	75.76	3072	2560	2560	2560
76.24	3072	2560	2560	3072	76.06	3072	2560	2560	3072
76.44	3584	2560	2560	3072	76.26	3584	2560	2560	3072
76.64	4096	2560	2560	3072	76.56	4096	2560	2560	3072
76.84	4608	2560	2560	3072	76.66	4608	2560	2560	3072
76.94	4608	3072	**2560**	3072	76.76	4608	3072	**2560**	3072
77.04	4608	3584	**2560**	3072	76.86	4608	3584	**2560**	3072
77.14	4608	3584	3072	3072	77.06	4608	3584	3072	3072
77.34	5120	3584	3584	**3072**	77.16	4608	3584	3584	**3072**
77.44	5120	3584	3584	3584	77.26	4608	3584	3584	3584
77.64	5120	4096	3584	3584	77.46	5120	3584	3584	3584
77.74	—	4096	3584	3584	77.56	—	4096	3584	3584
77.94	—	4608	4096	3584	77.86	—	5120	4096	3584
78.04	—	5120	4096	3584	78.06	—	5120	—	—
78.34	—	5120	—	—	78.20	—	5120	—	—
78.36	—	5120	—	—					

**Bolded** values indicate the minimum number of data samples required when smaller than in the other strategies. The symbol “—” denotes that the specified performance in the row was not achieved. Query strategies: RND = Random, LC = Least Confident, MRG = Margin, ENT = Entropy.

**Table A11:** Minimum number of training examples required by each Active Learning algorithm to reach the target ACC and F1 metrics on the DeepWeeds dataset using the EfficientNetB0 CNN.

ACC (%)	RND	LC	MRG	ENT	F1 (%)	RND	LC	MRG	ENT
70.23	—	—	—	—	69.77	—	—	—	—
70.33	512	512	512	512	69.87	512	512	512	512
70.43	1024	512	512	512	69.97	1024	512	512	512
71.23	1024	1024	512	512	70.77	1024	1024	512	512
71.33	1024	1024	**512**	1024	70.97	1024	1024	**512**	1024
71.43	1024	1024	1024	1024	71.17	1024	1024	1024	1024
76.23	1536	1024	1024	1024	75.77	1536	1024	1024	1024
76.93	1536	1024	1536	1024	76.67	1536	1024	1536	1024
77.03	1536	1536	1536	**1024**	76.77	1536	1536	1536	**1024**
78.03	1536	1536	1536	1536	77.77	1536	1536	1536	1536
78.93	2048	1536	1536	1536	78.47	2048	1536	1536	1536
79.93	2048	1536	1536	2048	79.67	2048	2048	**1536**	1536
80.03	2048	2048	**1536**	2048	79.77	2560	2048	**1536**	2048
80.13	2560	2048	**1536**	2048	80.17	2560	2048	2048	2048
80.43	2560	2048	2048	2048	80.67	3072	2048	2048	2048
80.93	3072	2048	2048	2048	81.37	3072	2048	2048	2560
81.63	3072	2048	2048	2560	81.77	3072	2048	2560	2560
82.03	3072	2048	2560	2560	81.87	3584	2048	2560	2560
82.13	3584	2048	2560	2560	82.17	3584	2560	2560	3072
82.33	3584	2560	2560	3072	82.87	4096	2560	2560	3072
83.03	4096	2560	2560	3072	83.17	4096	2560	3072	3072
83.33	4096	2560	3072	3072	83.27	4096	3072	3072	3072
83.53	4096	3072	3072	3072	83.77	4608	3072	3072	3072
84.03	5120	3072	3072	3072	83.97	5120	3072	3584	3584
84.13	5120	3072	3584	3584	84.07	5120	3584	3584	3584
84.33	5120	3584	3584	3584	84.37	—	3584	3584	3584
84.43	—	3584	3584	3584	84.47	—	4096	4096	3584
84.63	—	4096	3584	3584	84.67	—	4096	4608	4096
84.73	—	4096	4096	3584	84.87	—	4096	4608	4608
84.83	—	4096	4096	4096	84.97	—	—	—	—
84.93	—	4096	4608	4096					
85.03	—	4096	4608	4608					
85.13	—	4096	—	—					
85.14	—	—	—	—					

**Bolded** values indicate the minimum number of data samples required when smaller than in the other strategies. The symbol “—” denotes that the specified performance in the row was not achieved. Query strategies: RND = Random, LC = Least Confident, MRG = Margin, ENT = Entropy.

**Table A12:** Minimum number of training examples required by each Active Learning algorithm to reach the target ACC and F1 metrics on the DeepWeeds dataset using the ResNet50 CNN.

ACC (%)	RND	LC	MRG	ENT	F1 (%)	RND	LC	MRG	ENT
69.09	—	—	—	—	68.59	—	—	—	—
69.19	512	512	512	512	68.69	512	512	512	512
69.29	512	512	512	1024	68.79	512	512	512	1024
69.59	1024	512	1024	1024	69.19	1024	512	512	1024
70.49	1024	1024	1024	1024	69.29	1024	512	1024	1024
73.69	1024	1024	1024	1536	70.09	1024	1024	1024	1024
74.89	1536	1024	1024	1536	73.49	1024	1024	1024	1536
75.69	1536	1536	**1024**	1536	74.59	1536	1024	**1024**	1536
75.89	1536	1536	1536	1536	75.59	1536	1536	**1024**	1536
76.69	1536	1536	1536	2048	75.69	1536	1536	1536	1536
76.89	2048	1536	1536	2048	76.29	1536	1536	1536	2048
78.19	2048	2048	**1536**	2048	76.59	2048	1536	**1536**	2048
78.39	2048	2048	2048	2048	77.99	2048	2048	2048	2048
78.99	3072	2048	2048	2048	78.79	3072	2048	2048	2048
79.29	3072	2048	2048	2560	79.09	3072	2048	2048	2560
80.29	3072	2048	2560	2560	80.19	3072	2048	2560	2560
80.39	3072	2560	2560	2560	80.29	3072	2560	2560	2560
80.59	3584	2560	2560	2560	80.39	3584	2560	2560	2560
81.49	4096	2560	2560	2560	81.39	4096	2560	2560	2560
81.59	4096	3072	**2560**	2560	81.49	4096	3072	**2560**	3072
81.69	4096	3072	**2560**	3072	81.59	4096	3072	3072	3072
81.79	4608	3072	3072	3072	81.69	4608	3072	3072	3072
82.29	4608	3072	3072	3584	81.99	4608	3072	3072	3584
82.39	4608	3584	**3072**	3584	82.39	4608	3584	**3072**	3584
82.79	—	3584	4096	3584	82.59	4608	3584	4096	3584
83.29	—	4096	4096	3584	82.69	—	3584	4096	3584
83.69	—	4096	4608	3584	83.19	—	4096	4096	3584
83.79	—	4096	4608	4096	83.59	—	4096	4608	3584
83.89	—	4096	—	—	83.69	—	4096	4608	4096
84.03	—	—	—	—	83.79	—	4096	—	—
					83.87	—	—	—	—

**Bolded** values indicate the minimum number of data samples required when smaller than in the other strategies. The symbol “—” denotes that the specified performance in the row was not achieved. Query strategies: RND = Random, LC = Least Confident, MRG = Margin, ENT = Entropy.

## References

[1] Zhu Y., Wang M., Yin X., Zhang J., Meijering E., Hu J. (2022). Deep learning in diverse intelligent sensor based systems. Sensors.

[2] Lee S.H., Kang D.K. (2024). Deep Learning Technology and Image Sensing. Sensors.

[3] Vargas J., Alsweiss S., Toker O., Razdan R., Santos J. (2021). An overview of autonomous vehicles sensors and their vulnerability to weather conditions. Sensors.

[4] Yu X., Salimpour S., Queralta J.P., Westerlund T. (2023). General-purpose deep learning detection and segmentation models for images from a LiDAR-based camera sensor. Sensors.

[5] Cohen J.P., Morrison P., Dao L. (2020). COVID-19 image data collection. arXiv.

[6] Olsen A., Konovalov D.A., Philippa B., Ridd P., Wood J.C., Johns J., Banks W., Girgenti B., Kenny O., Whinney J. (2019). DeepWeeds: A multiclass weed species image dataset for deep learning. Sci. Rep..

[7] Li W., Gao Z., Song Y. (2025). U-ResNet, a Novel Network Fusion Method for Image Classification and Segmentation. Sensors.

[8] Mani P., Komarasamy P.R.G., Rajamanickam N., Shorfuzzaman M., Abdelfattah W.M. (2024). Enhancing sustainable transportation infrastructure management: A high-accuracy, FPGA-based system for emergency vehicle classification. Sustainability.

[9] Elgendy M. (2020). Deep Learning for Vision Systems.

[10] Tharwat A., Schenck W. (2023). A survey on active learning: State-of-the-art, practical challenges and research directions. Mathematics.

[11] Zhang S., Jafari O., Nagarkar P. (2021). A Survey on Machine Learning Techniques for Auto Labeling of Video, Audio, and Text Data. arXiv.

[12] Settles B. (2009). Active Learning Literature Survey.

[13] Settles B., Craven M. An analysis of active learning strategies for sequence labeling tasks. Proceedings of the 2008 Conference on Empirical Methods in Natural Language Processing, Association for Computational Linguistics.

[14] Németh G., Matuszka T. Compute-Efficient Active Learning. Proceedings of the NeurIPS 2023 Workshop on Adaptive Experimental Design and Active Learning in the Real World.

[15] Kim K., Jang S.J., Park J., Lee E., Lee S.S. (2023). Lightweight and energy-efficient deep learning accelerator for real-time object detection on edge devices. Sensors.

[16] Ahmad M., Zhang L., Chowdhury M.E. (2024). FPGA Implementation of Complex-Valued Neural Network for Polar-Represented Image Classification. Sensors.

[17] Bailey D.G. (2023). Design for Embedded Image Processing on FPGAs.

[18] Karamimanesh M., Abiri E., Shahsavari M., Hassanli K., van Schaik A., Eshraghian J. (2025). Spiking neural networks on FPGA: A survey of methodologies and recent advancements. Neural Netw..

[19] Li Z., Hong F.Z., Yue C.P. (2024). FPGA-based Acceleration of Neural Network for Image Classification using Vitis AI. arXiv.

[20] Dhilleswararao P., Boppu S., Manikandan M.S., Cenkeramaddi L.R. (2022). Efficient hardware architectures for accelerating deep neural networks: Survey. IEEE Access.

[21] Mouri Zadeh Khaki A., Choi A. (2025). Optimizing deep learning acceleration on FPGA for real-time and resource-efficient image classification. Appl. Sci..

[22] Lu A., Fan X., Tian J., Yang X. A Hardware-Software Co-Design FPGA Acceleration Platform for Driver Behavior Recognition Based on MobileNetV2. Proceedings of the 2025 IEEE 2nd International Conference on Deep Learning and Computer Vision (DLCV).

[23] Zhao M., Hu C., Wei F., Wang K., Wang C., Jiang Y. (2019). Real-time underwater image recognition with FPGA embedded system for convolutional neural network. Sensors.

[24] Pérez I., Figueroa M. (2021). A heterogeneous hardware accelerator for image classification in embedded systems. Sensors.

[25] Aydin S.G., Bilge H.Ş. (2023). Fpga implementation of image registration using accelerated cnn. Sensors.

[26] Xu Y., Luo J., Sun W. (2024). Flare: An FPGA-Based Full Precision Low Power CNN Accelerator with Reconfigurable Structure. Sensors.

[27] Ebrahim A. (2023). Finding the top-k heavy hitters in data streams: A reconfigurable accelerator based on an FPGA-optimized algorithm. Electronics.

[28] Ebrahim A., Khalifat J. (2023). Fast approximation of the top-k items in data streams using FPGAs. IET Comput. Digit. Tech..

[29] Kim R., Lee D., Kim J., Park J., Lee S.E. (2025). Hardware Accelerator for Approximation-Based Softmax and Layer Normalization in Transformers. Electronics.

[30] Gaillochet M., Desrosiers C., Lombaert H. (2023). Active learning for medical image segmentation with stochastic batches. Med. Image Anal..

[31] Zhao Z., Zeng Z., Xu K., Chen C., Guan C. (2021). Dsal: Deeply supervised active learning from strong and weak labelers for biomedical image segmentation. IEEE J. Biomed. Health Inform..

[32] Ge J., Zhang Z., Phan V.M.H., Zhang B., Liu A., Zhao Y., Zhao S. (2025). Esa: Annotation-efficient active learning for semantic segmentation. Proceedings of the 21st International Conference, ICIC 2025, Ningbo, China, 26–29 July 2025.

[33] Yu W., Zhu S., Yang T., Chen C. Consistency-based active learning for object detection. Proceedings of the IEEE/CVF Conference on Computer Vision and Pattern Recognition.

[34] Li Y., Fan B., Zhang W., Ding W., Yin J. (2021). Deep active learning for object detection. Inf. Sci..

[35] Jiu M., Song X., Sahbi H., Li S., Chen Y., Guo W., Guo L., Xu M. (2024). Image classification with deep reinforcement active learning. arXiv.

[36] Hacohen G., Dekel A., Weinshall D. Active Learning on a Budget: Opposite Strategies Suit High and Low Budgets. Proceedings of the International Conference on Machine Learning, PMLR.

[37] Khaleel M., Idris A., Tavanapong W., Pratt J.R., Oh J., C. de Groen P. (2023). VisActive: Visual-concept-based active learning for image classification under class imbalance. ACM Trans. Multimed. Comput. Commun. Appl..

[38] Wang Z., Chen Z., Du B. (2023). Active learning with co-auxiliary learning and multi-level diversity for image classification. IEEE Trans. Circuits Syst. Video Technol..

[39] Abdelwahab A., Afifi A., Salama M. (2023). An integrated active deep learning approach for image classification from unlabeled data with minimal supervision. Electronics.

[40] Griffin G., Holub A., Perona P. (2022). Caltech 256 (1.0); Data set. https://data.caltech.edu/records/nyy15-4j048.

[41] Gashi E., Deng J., Elezi I. (2025). Deep active learning: A reality check. Pattern Recognit. Lett..

[42] Krizhevsky A., Hinton G. (2009). Learning Multiple Layers of Features from Tiny Images.

[43] Li F.F., Andreeto M., Ranzato M., Perona P. (2022). Caltech 101 (1.0); Data set. https://data.caltech.edu/records/mzrjq-6wc02.

[44] Shen Y., Song Y., Wu C.h., Kuo C.C.J. (2022). TBAL: Two-stage batch-mode active learning for image classification. Signal Process. Image Commun..

[45] Vununu C., Lee S.H., Kwon K.R. (2021). A classification method for the cellular images based on active learning and cross-modal transfer learning. Sensors.

[46] Chen Y., Wen Z., Biros G. A Scalable Algorithm for Active Learning. Proceedings of the SC24: International Conference for High Performance Computing, Networking, Storage and Analysis, IEEE.

[47] Yang C., Huang L., Crowley E.J. Plug and play active learning for object detection. Proceedings of the IEEE/CVF Conference on Computer Vision and Pattern Recognition.

[48] Simonyan K., Zisserman A. Very deep convolutional networks for large-scale image recognition. Proceedings of the 3rd International Conference on Learning Representations (ICLR 2015), Computational and Biological Learning Society.

[49] Sandler M., Howard A., Zhu M., Zhmoginov A., Chen L.C. Mobilenetv2: Inverted residuals and linear bottlenecks. Proceedings of the IEEE Conference on Computer Vision and Pattern Recognition.

[50] Montoya A., Holman D., Science S.D., Smith T., Kan W. (2016). State Farm Distracted Driver Detection. https://kaggle.com/competitions/state-farm-distracted-driver-detection.

[51] Duarte J., Han S., Harris P., Jindariani S., Kreinar E., Kreis B., Ngadiuba J., Pierini M., Rivera R., Tran N. (2018). Fast inference of deep neural networks in FPGAs for particle physics. J. Instrum..

[52] Umuroglu Y., Fraser N.J., Gambardella G., Blott M., Leong P., Jahre M., Vissers K. FINN: A Framework for Fast, Scalable Binarized Neural Network Inference. Proceedings of the 2017 ACM/SIGDA International Symposium on Field-Programmable Gate Arrays, ACM, FPGA’17.

[53] Blott M., Preußer T.B., Fraser N.J., Gambardella G., O’brien K., Umuroglu Y., Leeser M., Vissers K. (2018). FINN-R: An end-to-end deep-learning framework for fast exploration of quantized neural networks. ACM Trans. Reconfigurable Technol. Syst. (TRETS).

[54] Reddy B.N.K., Sarangam K., Dandeliya S., Naidu S.P.S., Kumar N. Accelerating sorting performance on FpGa: Combining Quick sort and Heap sort through Hybrid pipelining. Proceedings of the 2023 IEEE International Symposium on Smart Electronic Systems (iSES).

[55] Qiao W., Guo L., Fang Z., Chang M.C.F., Cong J. (2022). TopSort: A high-performance two-phase sorting accelerator optimized on HBM-based FPGAs. IEEE Trans. Emerg. Top. Comput..

[56] Sun M., Xie G., Zhang F., Guo W., Fan X., Chen L., Du J. (2025). FPGA-Based Large-Scale Sorting with Optimized Bandwidth Utilization. ACM Trans. Reconfigurable Technol. Syst..

[57] Ji Y., Wei D., Shi L., Liu P., Li C., Yu J., Cai X. An Active Learning based Latency Prediction Approach for Neural Network Architecture. Proceedings of the 4th International Conference on Neural Networks, Information and Communication Engineering (NNICE), IEEE.

[58] Sun Q., Bai C., Geng H., Yu B. Deep neural network hardware deployment optimization via advanced active learning. Proceedings of the 2021 Design, Automation & Test in Europe Conference & Exhibition (DATE), IEEE.

[59] Ghaffari A., Asgharian M., Savaria Y. (2023). Statistical hardware design with multimodel active learning. IEEE Trans. Comput.-Aided Des. Integr. Circuits Syst..

[60] Chen W., Li W., Yu F. (2019). A hybrid pipelined architecture for high performance top-K sorting on FPGA. IEEE Trans. Circuits Syst. II Express Briefs.

[61] Ben-Jmaa Y., Duvivier D. (2024). Sorting Algorithms Comparison on FPGA and Intel i7 Architectures. Comput. Y Sist..

[62] Ben Jmaa Y., Ben Atitallah R., Duvivier D., Ben Jemaa M. (2019). A comparative study of sorting algorithms with FPGA acceleration by high level synthesis. Comput. Y Sist..

[63] Xiao H., Rasul K., Vollgraf R. (2017). Fashion-mnist: A novel image dataset for benchmarking machine learning algorithms. arXiv.

[64] Bouguezzi S., Fredj H.B., Belabed T., Valderrama C., Faiedh H., Souani C. (2021). An efficient FPGA-based convolutional neural network for classification: Ad-MobileNet. Electronics.

[65] Nocentini O., Kim J., Bashir M.Z., Cavallo F. (2022). Image classification using multiple convolutional neural networks on the fashion-MNIST dataset. Sensors.

[66] Kim Y.J. (2022). Machine learning model based on radiomic features for differentiation between COVID-19 and pneumonia on chest X-ray. Sensors.

[67] Murad N.Y., Mahmood T., Forkan A.R.M., Morshed A., Jayaraman P.P., Siddiqui M.S. (2023). Weed detection using deep learning: A systematic literature review. Sensors.

[68] Howard A.G., Zhu M., Chen B., Kalenichenko D., Wang W., Weyand T., Andreetto M., Adam H. (2017). MobileNets: Efficient Convolutional Neural Networks for Mobile Vision Applications. arXiv.

[69] Tan M., Le Q., Chaudhuri K., Salakhutdinov R. EfficientNet: Rethinking Model Scaling for Convolutional Neural Networks. Proceedings of the 36th International Conference on Machine Learning (PMLR), Long Beach, CA, USA, 9–15 June 2019.

[70] He K., Zhang X., Ren S., Sun J. Deep residual learning for image recognition. Proceedings of the 2016 IEEE Conference on Computer Vision and Pattern Recognition (CVPR).

[71] Russakovsky O., Deng J., Su H., Krause J., Satheesh S., Ma S., Huang Z., Karpathy A., Khosla A., Bernstein M. (2015). ImageNet Large Scale Visual Recognition Challenge. Int. J. Comput. Vis. (IJCV).

[72] Intel Intel Xeon Processor E5 v4 Family. Intel ARK (Product Specifications), 2025. https://www.intel.com/content/www/us/en/ark/products/series/91287/intel-xeon-processor-e5-v4-family.html.

[73] Askar T., Yergaliyev A., Shukirgaliyev B., Abdikamalov E. (2024). Exploring Numba and CuPy for GPU-Accelerated Monte Carlo Radiation Transport. Computation.

[74] Malinin A., Gales M. (2018). Predictive uncertainty estimation via prior networks. Adv. Neural Inf. Process. Syst..

[75] Cho M., Kim Y. (2021). FPGA-based convolutional neural network accelerator with resource-optimized approximate multiply-accumulate unit. Electronics.

[76] Xu J., Pu H., Wang D. (2024). Sparse Convolution FPGA Accelerator Based on Multi-Bank Hash Selection. Micromachines.

[77] Al Amin R., Hasan M., Wiese V., Obermaisser R. (2024). FPGA-based real-time object detection and classification system using YOLO for edge computing. IEEE Access.

[78] Reddy D.V., Rao M.N., Satyanarayana T., Babu T.A., Reddy K.V.V. (2025). A lightweight FPGA Accelerator for Onboard Processing of Hyperspectral Anomaly Detection based on Optimized TinyYOLOv3 Model. Integration.

[79] Moreira C.M., Shneider C., Hein A.M. (2025). Edge computing in space: Design of an FPGA architecture for thermal anomaly detection based on a machine learning approach. Adv. Space Res..

